# Molecular Characterization and Expression Profiling of Tomato GRF Transcription Factor Family Genes in Response to Abiotic Stresses and Phytohormones

**DOI:** 10.3390/ijms18051056

**Published:** 2017-05-13

**Authors:** Khadiza Khatun, Arif Hasan Khan Robin, Jong-In Park, Ujjal Kumar Nath, Chang Kil Kim, Ki-Byung Lim, Ill Sup Nou, Mi-Young Chung

**Affiliations:** 1 Department of Agricultural Industry Economy and Education, Sunchon National University, 413 Jungangno, Suncheon, Jeonnam 540-950, Korea; kkr@pstu.ac.bd; 2 Department of Horticulture, Sunchon National University, 413 Jungangno, Suncheon, Jeonnam 540-950, Korea; gpb21bau@bau.edu.bd (A.H.K.R.); jipark@sunchon.ac.kr (J.-I.P.); ujjalnath@gmail.com (U.K.N.); nis@sunchon.ac.kr (I.S.N.); 3 Department of Horticultural Science, Kyungpook National University, Daegu 702-701, Korea; ckkim@knu.ac.kr (C.K.K.); kblim@knu.ac.kr (K.-B.L.); 4 Department of Agricultural Education, Sunchon National University, 413 Jungangno, Suncheon, Jeonnam 540-950, Korea

**Keywords:** *GRF* gene, *Solanum lycopersicum*, organ-specific expression, fruit development, abiotic stress, phytohormone treatment

## Abstract

Growth regulating factors (GRFs) are plant-specific transcription factors that are involved in diverse biological and physiological processes, such as growth, development and stress and hormone responses. However, the roles of GRFs in vegetative and reproductive growth, development and stress responses in tomato (*Solanum lycopersicum*) have not been extensively explored. In this study, we characterized the 13 *SlGRF* genes. In silico analysis of protein motif organization, intron–exon distribution, and phylogenetic classification confirmed the presence of GRF proteins in tomato. The tissue-specific expression analysis revealed that most of the *SlGRF* genes were preferentially expressed in young and growing tissues such as flower buds and meristems, suggesting that *SlGRFs* are important during growth and development of these tissues. Some of the *SlGRF* genes were preferentially expressed in fruits at distinct developmental stages suggesting their involvement in fruit development and the ripening process. The strong and differential expression of different *SlGRFs* under NaCl, drought, heat, cold, abscisic acid (ABA), and jasmonic acid (JA) treatment, predict possible functions for these genes in stress responses in addition to their growth regulatory functions. Further, differential expression of *SlGRF* genes upon gibberellic acid (GA3) treatment indicates their probable function in flower development and stress responses through a gibberellic acid (GA)-mediated pathway. The results of this study provide a basis for further functional analysis and characterization of this important gene family in tomato.

## 1. Introduction

Transcription factors regulate many biological processes in plants such as growth, development, metabolism, reproduction and differentiation [1]. Growth-regulating factors (GRFs) are small plant-specific transcription-factors involved in the regulation of plant growth, development and longevity [2,3,4,5]. The first *GRF* gene was identified in rice (*Oryza sativa*) where it mediates gibberellic acid (GA)-induced regulation of stem growth in deepwater rice [6]. GRF family members are characterized by two conserved domains, QLQ (Gln, Leu, Gln) and WRC (Trp, Arg, Cys), in the N-terminal region [6,7,8]. The QLQ domain is also found in SWI2/SNF2 (SWItch/Sucrose non-fermentable) protein, which is a chromatin-remodeling protein complex in *Saccharomyces cerevisiae* [9]. QLQ acts as a transcriptional co-activator in protein–protein interactions through the interaction with GRF-interacting factors (GIFs) [10,11]. By contrast, WRC is a plant-specific domain that is capable of binding to DNA and possesses a functional nuclear localization signal and a zinc-finger motif (C_3_H) [8]. In addition to the QLQ and WRC domains, GRF proteins contain less-conserved TQL ((Thr, Gln, Leu), FFD (Phe, Phe, Asp) and GGPL domains in their C-terminal regions [6,8,11]. To date, the *GRF* gene family comprises 9 members in *Arabidopsis*, 12 in rice, 14 in maize and 17 in Chinese cabbage (*Brassica rapa*) [7,8,10,11,12]. Several studies have reported that the expression of *GRF* genes in actively growing and developing tissue is higher than in mature tissue [7,8,11,12]. *GRF* genes regulate leaf size and shape, and the development of the shoot apical meristem and cotyledons through stimulation of cell proliferation [10,13]. GRF genes also regulate flower development [14,15,16] and stimulate oil production in rapeseed [17]. In addition, GRFs function in female reproductive organ development and ovule formation in *Arabidopsis* [18]. Expression of GRFs has also been observed in various rice and maize tissues [7,19]. These results suggest the possible involvement of GRFs in the development of seed. Some *GRF* genes suppress expression of Knotted1-like Homeobox (*KNOX*) genes, which inhibit cell differentiation in the shoot apical meristem [20]. The activity and abundance of GRF transcripts are inhibited by the miR396 microRNA which appeared to control seven of the nine genes in *Arabidopsis* [3]. During the formation of syncytia (multinucleated cells formed through multiple cell fusions of uninuclear cells) upon nematode infection, the miR396-GRF regulatory modules act as developmental regulators that reduce syncytium size and arrest nematode development in *Arabidopsis* [21]. Transcription factors regulate the expression of target genes in response to growth, development and environmental stresses through various signaling networks [22]. In *Arabidopsis*, AtGRF7 acts as a repressor of osmotic stress-responsive genes under normal conditions to minimize the adverse effects of those genes on plant growth, while under stress conditions its expression is inhibited to activate osmotic stress-responsive genes [23]. Potential involvement of *AtGRF1* and *AtGRF3* genes was found in the biotic stress response after infection with the *Heterodera schachtii* nematode [21]. However, for many plant species, the role of plant *GRF* family genes in stress responses remains to be revealed.

Genome-wide identification and characterization of the *GRF* transcription factor genes has been completed in *Arabidopsis* and a number of crops such as rice, Chinese cabbage and maize [7,8,11,12]. Cao et al. (2016) reported approximately 13 *GRF* family genes in tomato but their involvement in growth, development and defense-related activities was not extensively studied in tomato [24]. Here, we systematically characterized all 13 putative *GRF* family genes of tomato and carried out an evolutionary analysis of tomato GRF genes by comparing them with those of *Arabidopsis*, rice, maize and Chinese cabbage. We also analyzed the expression profiles of tomato *GRF* genes in different tissues and at different stages of fruit development. Furthermore, the expression patterns of tomato *GRF* genes were analyzed under different abiotic stress conditions. Finally, GA-responsive expression patterns of tomato *GRF* genes were examined.

## 2. Results

### 2.1. Identification and Sequence Analysis of SlGRF Genes and Their Putative Proteins

**Figure 1:**
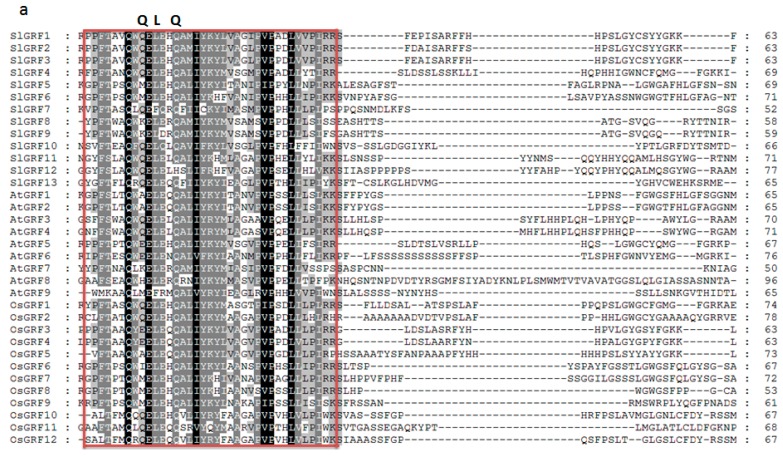

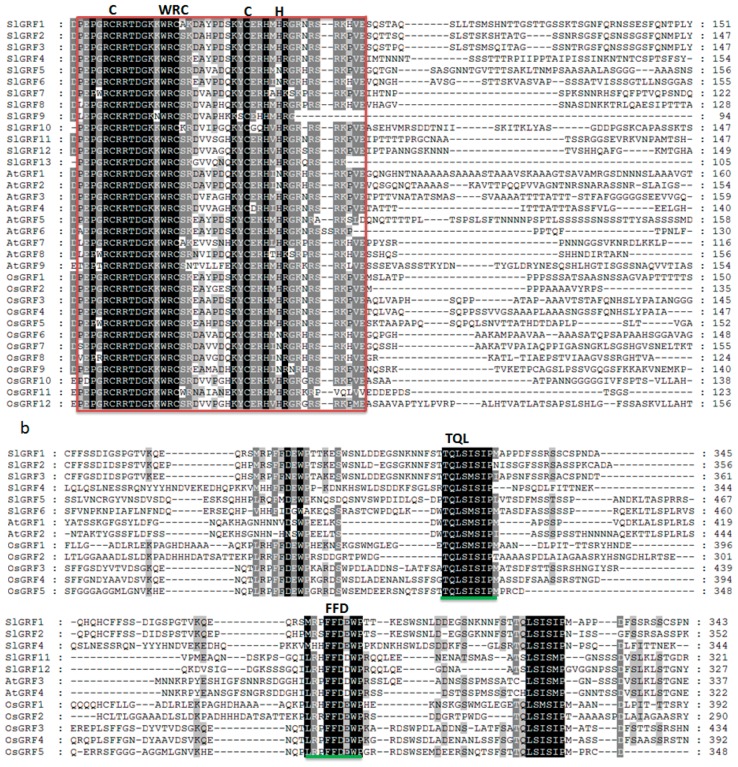
Sequence alignment of SlGRF (*Solanum lycopersicum* GRF) proteins and GRF proteins from *Arabidopsis* and rice: (**a**) the QLQ and WRC domains are indicated by the red box; and (**b**) the TQL and FFD motifs are green underlined. Identical amino acids are indicated by black and the amino acids with >50% similarity is indicated by gray background.

Thirteen tomato genes were identified that potentially encode GRF proteins. These 13 tomato *GRF* genes were designated SlGRF1–SlGRF13 according to Cao et al. (2016). Considerable variation in length was found in the coding DNA sequence (CDS) of *SlGRF*s: from 468 to 1788 bp for *SlGRF9* and *SlGRF5*, respectively (Table 1). The length of the identified tomato GRF proteins ranged from 155 to 595 amino acids (aa), the molecular weight (MW) ranged from 17.3958 to 64.118 kDa, and the iso-electric point (pI) ranged from 5.97 to 9.18. All of the putative SlGRF proteins had both QLQ and WRC domains in the N-terminal region (Table 1, Figure 1a, Figure S1). SlGRF10 also contained a second WRC domain downstream of the first one (Figure S1). A zinc finger motif (CCCH) was also found within the WRC domain in all SlGRF proteins (Figure 1a). The SlGRF1, SlGRF2, SlGRF3, SlGRF4, SlGRF5, and SlGRF6 proteins shared a short stretch of amino acid residues termed the TQL domain, and SlGRF1, SlGRF2, SlGRF4, SlGRF11, and SlGRF12 shared a FFD domain in their C-terminal regions (Figure 1b, Figure S1). The QLQ domain involved in protein–protein interactions and the WRC domain act as DNA-binding domain with a putative nuclear localization signal and the C-terminal TQL, FFD motifs may be served as transactivation domain [5,6,7]. The C-terminal region of tomato GRF proteins was rich in acidic amino acids, namely aspartic acid (D) and glutamic acid (E) (Figure 1a,b).

### 2.2. Evolutionary Analysis of SlGRF Proteins through Phylogenetic Classification

**Figure 2:**
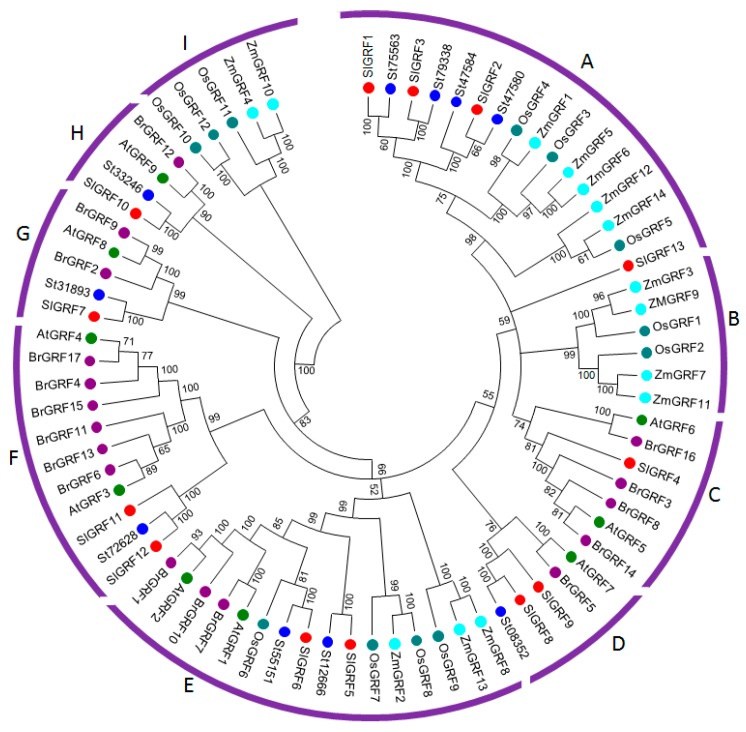
Phylogenetic analysis of GRF proteins from tomato, potato (St, *Solanum tuberosum* is used instead of PGSC0003DMT), *Arabidopsis* (*Arabidopsis thaliana* GRF, AtGRF), rice (*Oryza sativa* GRF, OsGRF), maize (*Zea mays* GRF-ZmGRF) and Chinese cabbage (*Brassica rapa* GRF, BrGRF). The phylogenetic tree was established with entire protein sequences from the above plant species by the UPGMA (Unweighted Pair Group Method with Arithmetic mean) method following the pair-wise deletion method. The numbers on the branches indicate bootstrap support values from 1000 replications. The scale represents the units of the number of amino acid substitutions per site. The protein sequences used in the phylogenetic analysis are listed in Additional File 1 with their accession IDs.

To establish the evolutionary and functional relationships within the GRF family, a phylogenetic tree was constructed of the 13 putative tomato GRFs, 10 potato GRF, 9 *Arabidopsis* GRFs, 12 rice GRFs, 14 maize GRFs, and 17 Chinese cabbage GRF proteins (Figure 2). The phylogenetic tree classified the 75 GRF proteins into nine subgroups (A–I) based on clade and evolution of species in the topology of the trees. Subgroups B and I contained GRFs from monocot species only, whereas subgroups C, D, F, G and H contained GRFs from dicot species only. Notably, subgroups A and E contained clusters of GRFs from both monocot and dicot species. The 13 tomato GRF were distributed into seven of nine subgroups except for the subgroup B and I. The highest number of SlGRFs was clustered in subgroups A containing four members: SlGRF1, SlGRF2, SlGRF3 and SlGRF13. Subgroups E and F each contained two SlGRFs (SlGRF5 and SlGRF6 in subgroup E, and SlGRF11 and SlGRF12 in subgroup F).

### 2.3. Structural Organization, and Chromosomal Location of SlGRF Genes

**Figure 3:**
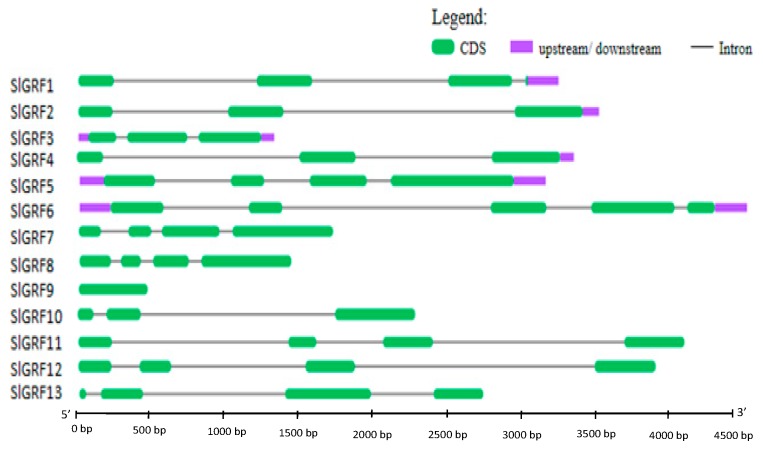
Exon–intron distribution of *SlGRF* genes. Exons and introns are represented by green boxes and black lines, respectively.

With the exception of *SlGRF9*, all *SlGRF* genes contained introns in their coding sequences (Figure 3). The number of introns varied from two to four: most of the genes (7 out of 12) contained three introns, four genes contained two introns, and one gene contained four introns.

The thirteen *SlGRF* genes were distributed over 9 of the 12 tomato chromosomes, with five of the genes found on chromosome 8 (Figure S2). Chromosomes 1, 2, 3, 4, 7, 9, 10 and 12 each contained a single *SlGRF* gene. No segmental or tandem duplication was found among the 13 *SlGRF* genes, suggesting that gene duplication did not play a role in expansion of the *GRF* gene family in tomato (Figure S2, Table S3).

### 2.4. Putative Cis-Elements and Functional Analysis of SlGRF Genes

From promoter analysis, we found that with the exception of *SlGRF5*, *SlGRF10* and *SlGRF11*, all other *SlGRF* genes contained gibberellin-responsive *cis*-regulatory elements in their promoter regions (Table S4). In addition, several other cis-acting elements that were related to tissue-specific expression, development, auxin and ethylene response, circadian regulation, and abiotic and biotic stress response were found in the promoter regions of *SlGRF* genes. These findings imply that tomato *GRF* family genes could function in development and stress tolerance.

Analysis of putative functions based gene ontology (GO) classifications placed all 13 SlGRF proteins in some similar and common groups including: adenosine triphosphate (ATP) binding in the molecular function category, regulation of transcription in the biological process category, and nucleus in the cellular component category (Table S5). Although the 13 *GRF* genes have some common functions based on GO category, however, due to the variation of *cis*-elements in promoter regions, there might have some functional variation among the genes.

### 2.5. Expression Analysis of Tomato GRF Genes in Different Tissues

**Figure 4:**
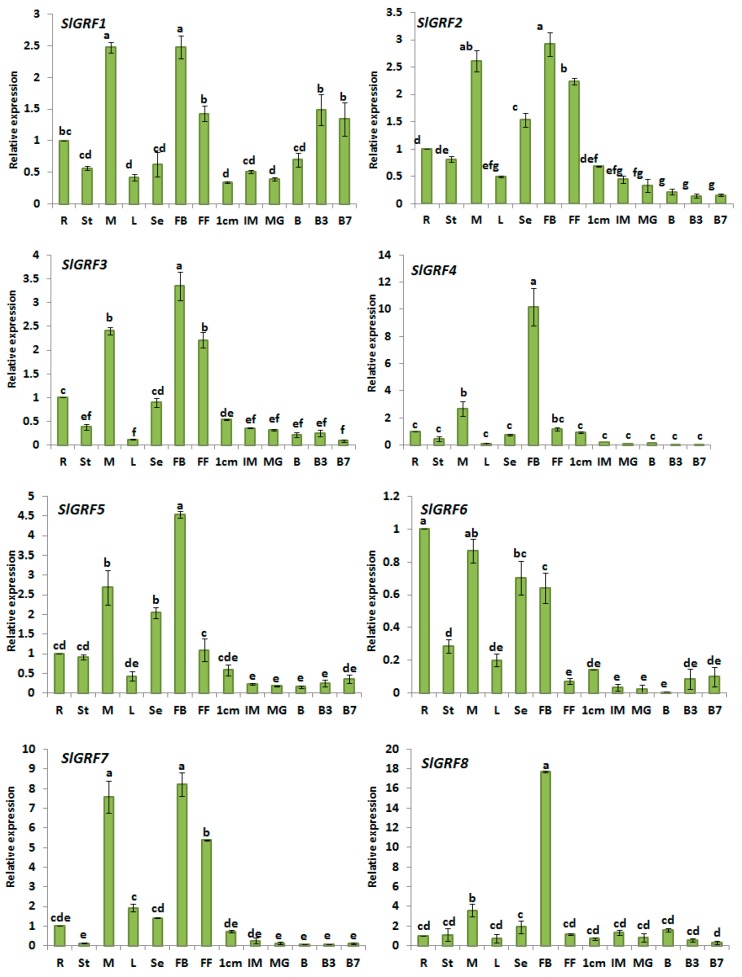

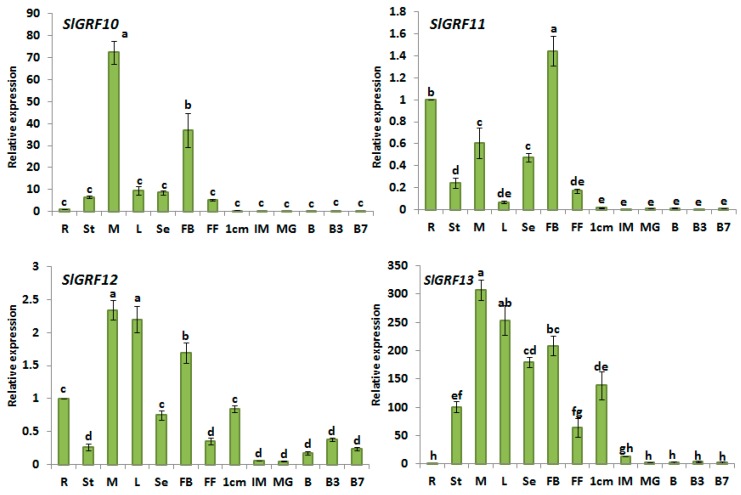
Expression of *SlGRF* genes in different organs. Root (R), stem (St), meristem (M), leaves (L), seedling (Se), flower bud (FB), full blooming flower (FF), and fruits at six developmental stages (1 cm: 1 centimeter-sized fruit, IM: immature fruit, MG: mature green fruit, B: breaker, B3: three days after breaker, B7: seven days after breaker) were analyzed by quantitative reverse transcription polymerase chain reaction (qRT-PCR). Relative gene expression levels are normalized to *EF1a* (*Elongation factor 1a*) values. Error bars represent standard deviations of the means of three independent replicates. Statistically significant variations of expression and mean values at different sampling points (ANOVA, *p* ≤ 0.01 for all 12 genes) are indicated with different letters.

The tissue-specific expression profiling of a gene family can provide clues about its possible functional roles in developmental processes. From our expression analysis of *SlGRF* genes it was evident that most of the genes showed tissue-specific expression patterns. For example, *SlGRF1*, *SlGRF2*, *SlGRF3*, *SlGRF4*, *SlGRF5*, *SlGRF7*, and *SlGRF8* were preferentially expressed in flower buds (2.5 to 18-fold versus root samples) followed by vegetative meristem (2.0–7-fold versus root samples) compared to other vegetative tissues (e.g., roots, stems, and leaves) and developing fruits (Figure 4). Among these genes, *SlGRF4* and *SlGRF8* exhibited 10- and 18-fold higher expression, respectively, in flower buds compared to root tissues (control). *SlGRF10* was most abundantly expressed in meristem, 70-fold higher than control, followed by flower bud, 35-fold higher than control, compared to other vegetative tissues (e.g., roots, stems, and leaves) and developing fruits. We identified only one gene, *SlGRF6*, that showed the highest level of transcript accumulation in root. In addition, we identified one gene, *SlGRF12*, that had the highest expression in meristem and leaves. *SlGRF13* was expressed between 50- and 300-fold more highly in vegetative tissues and flower organs compared to root samples. This gene also showed 140-fold higher expression in 1 cm fruit compared to root samples.

In general, most of the *SlGRF* genes were more highly expressed in vegetative tissues and flower organs compared to developing fruits (Figure 4). The expression of *SlGRF2*, *SlGRF3*, *SlGRF5*, *SlGRF12* and *SlGRF13* was high in small green fruit (1 cm fruit) relative to that in the mature and ripening fruit (Figure 4). The expression of *SlGRF1* increased during fruit ripening after the breaker stage compared with 1 cm fruit, immature and mature green fruits. Expression of *SlGRF10* and *SlGRF11* declined from 72- to 463-fold in developing fruits compared to flower buds (Figure 4).

RAN-sequencing (RNA-Seq) data obtained from the Solgenomics database were generally consistent with the expression pattern revealed by quantitative reverse transcription polymerase chain reaction (qRT-PCR) data from this study (Figure 4, Figure S3). For instance, *SlGRF3*, *SlGRF5*, *SlGRF8*, *SlGRF10* and *SlGRF12* are highly expressed in young flower bud followed by meristem. However, there were a few dissimilarities between expression results obtained from qRT-PCR and RNA-Seq methods. For example, *SlGRF6* was preferentially expressed in the root in this study (Figure 4) but RNA-Seq data reported its higher expression in meristem (Figure S3).

From the phylogenetic analysis, we found that only some members within the same phylogenetic subfamily shared a similar expression profile in tomato organs/tissue while other show different expression profiles. For instance, *SlGRF1*, *SlGRF2* and *SlGRF3* belonging to subfamily A showed higher expression in flower bud while another member, *SlGRF13*, was highly expressed in meristem. *SlGRF5* and *SlGRF6* belonging to subfamily E also showed divergence in expression patterns in different tissues. However, most of the tomato *GRF* genes within the same subfamily in the phylogenetic tree have similar *cis*-acting elements in their promoter regions (Table S4, Figure S2).

### 2.6. Expression Analysis of Tomato GRF Genes under Different Abiotic Stresses

To elucidate whether *SlGRFs* play roles under different abiotic stress conditions and in response to phytohormones, the expression patterns of eleven *SlGRF* genes (*SlGRF1*, *SLGRF2*, *SlGRF3*, *SlGRF4*, *SlGRF5*, *SlGRF6*, *SlGRF7*, *SlGRF10*, *SlGRF11*, *SlGRF12*, *SlGRF13*) in the leaf samples were determined by qRT-PCR. *SlGRF8* and *SlGRF9* did not show detectable expression under any of the abiotic stresses or phytohormone treatments. We monitored the expression of genes during a 24-h treatment period with measurements at 0 h, 1 h, 3 h, 9 h and 24 h, and compared the expression levels with control (0 h) samples.

#### 2.6.1. NaCl Treatment

**Figure 5:**
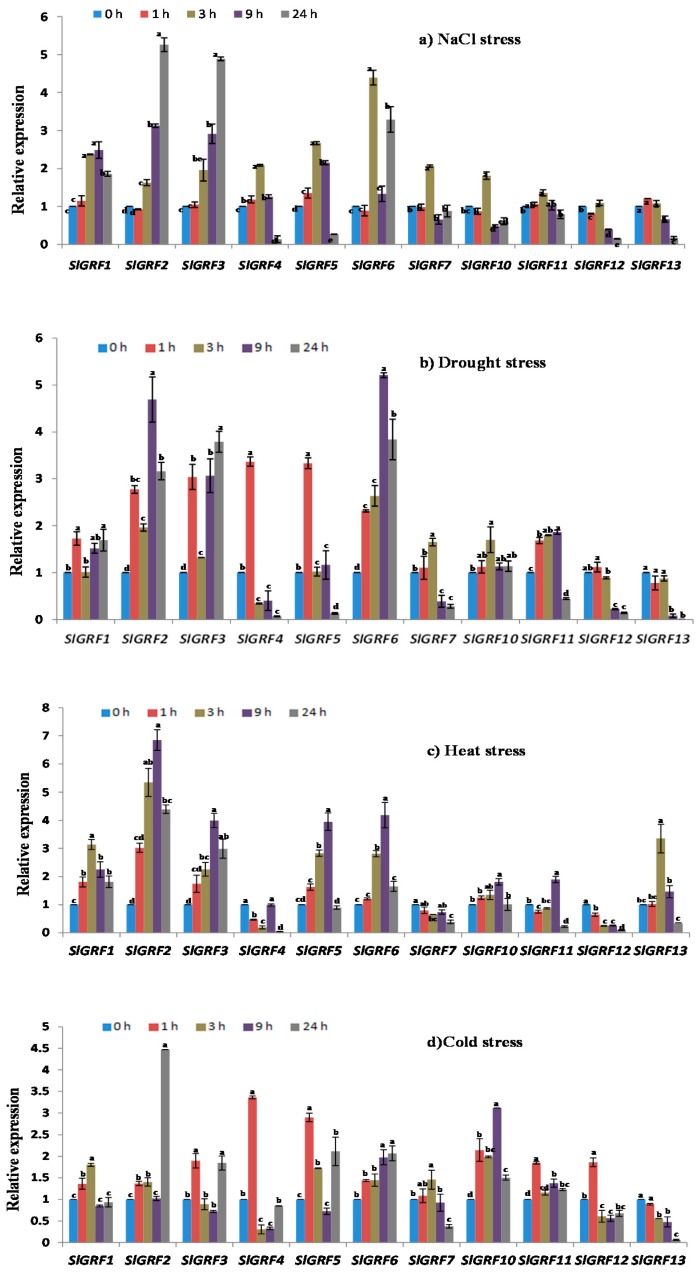
Expression of *SlGRF* genes in response to abiotic stresses: (**a**) NaCl; (**b**) drought; (**c**) heat; and (**d**) cold, at 0–24 h. The error bars represent the standard error of the means of three independent replicates of qRT-PCR analysis. Different letters associated with each treatment indicate statistically significant difference at 5% level of significance, where the same letter indicates that the values did not differ significantly at *p* ≤ 0.05 according to Tukey’s pairwise comparison tests.

Of the eleven studied *SlGRF* genes, *SlGRF1*, *SlGRF2*, *SlGRF3*, *SlGRF4*, *SlGRF5*, *SlGRF6* and *SlGRF7* showed significantly different expression levels (≥2-fold change) at different time points under NaCl stress compared to control—0 h after commencement of treatment—(Figure 5a). *SlGRF2* and *SlGRF3* showed approximately 4.5-fold increased expression at 24 h after treatment, and *SlGRF1* showed approximately 2.5-fold increased expression at 3 and 9 h after treatment, compared with the control (*p* ≤ 0.05, Figure 5a). *SlGRF4* was significantly up-regulated at 3 h after treatment compared with the control (*p* ≤ 0.05, Figure 5a). *SlGRF5* exhibited comparatively higher transcript levels at 3 and 9 h after treatment, whereas *SlGRF6* had higher transcript levels at 3 and 24 h after treatment compared to control (*p* ≤ 0.05, Figure 5a). The expression of *SlGRF5* and *SlGRF7* was increased from 1.5- to 2.5-fold at 3 h after treatment compared to the control (*p* ≤ 0.05, Figure 5a). With regard to the other genes, *SlGRF10*, *SlGRF11* and *SlGRF12* showed significantly higher expression at 3 h after treatment compared control (Figure 5a).

#### 2.6.2. Drought Treatment

The *SlGRF* genes showed differential expression at different time points under drought stress compared to the control (Figure 5b). *SlGRF1* had higher expression of between 1.5- and 2.0-fold at 1, 9 and 24 h after treatment compared to the control. *SlGRF2*, *SlGRF3* and *SlGRF6* showed upregulation of between 1.5- and 5.0-fold from 1 to 24 h after treatment compared with the control (*p* ≤ 0.05, Figure 5b). The expression patterns of *SlGRF4* and *SlGRF5* sharply increased at 1 h after treatment, where *SlGRF7* and *SlGRF10* increased at 3 h after treatment compared with the control (Figure 5b). The expression of *SlGRF11* increased between 1.5- and 2.0-fold from 1 to 9 h after treatment compared the control (*p* ≤ 0.05, Figure 5b). Interestingly, *SlGRF12* and *SlGRF13* expression was markedly downregulated from 4.5- to 12-fold from 9 to 24 h after treatment in comparison to the control (Figure 5b).

#### 2.6.3. Heat Treatment

*SlGRF1*, *SlGRF2*, *SlGRF3*, *SlGRF5* and *SlGRF6* were markedly up-regulated showing 1.5- to 6-fold increased expression, whereas *SlGRF7* and *SlGRF12* were down-regulated at all time points during the heat treatment period compared with the control (*p* ≤ 0.05, Figure 5c). The expression of *SlGRF4* was downregulated, except at 9 h after treatment, in comparison to control (*p* ≤ 0.05, Figure 5c). *SlGRF10* was upregulated from 1.2- to 2.0-fold at 3 and 9 h after treatment compared to control. The expression of *SlGRF11* was highly up-regulated at 9 h after treatment compared to control (*p* ≤ 0.05, Figure 5c). An approximate three-fold increase in expression of *SlGRF13* was found at 3 h after treatment compared to the control (*p* ≤ 0.05, Figure 5c).

#### 2.6.4. Cold Treatment

Notable variation in expression patterns of the eleven *SlGRF* genes was observed at different time points during cold treatment (Figure 5d). *SlGRF1* expression increased only at 1 and 3 h after treatment in comparison to the control (*p* ≤ 0.05, Figure 5d). The expression of *SlGRF2* was 4 fold increased only at 24 h after treatment. Expression of *SlGRF3* increased between 1.5 and 2-fold at 1 and 24 h after treatment, whereas that of *SlGRF5* increased between 1.5- and 3-fold at 1, 3 and 24 h after treatment compared to control (*p* ≤ 0.05, Figure 5d). *SlGRF6*, *SlGRF10* and *SlGRF11* were up-regulated approximately 1.5- to 3-fold at all time points compared to the control (*p* ≤ 0.05, Figure 5d). Higher expression levels, from 2 to 3-fold of *SlGRF4* and *SlGRF12* were detected at 1 h after treatment in comparison with the control (*p* ≤ 0.05, Figure 5d). Expression of *SlGRF13* was down-regulated more than 1-fold at 24 h after treatment compared to the control.

### 2.7. Expression Profiles of SlGRF Genes in Response to Phytohormone Treatments

#### 2.7.1. GA3 Treatment

**Figure 6:**
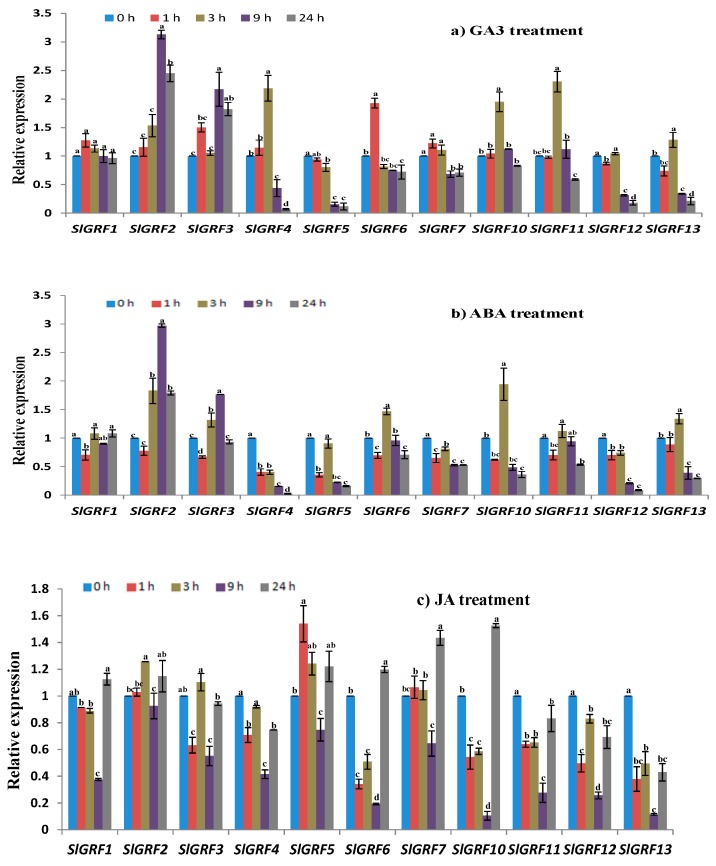
Expression of *SlGRF* genes in response to phytohormone treatments: (**a**) gibberellic acid (GA3); (**b**) abscisic acid (ABA); and (**c**) jasmonic acid (JA) treatment at 0–24 h. The error bars represent the standard error of the means of three independent replicates of qRT-PCR analysis. Different letters associated with each treatment indicate statistically significant difference at 5% level of significance, where the same letter indicates that the values did not differ significantly at *p* ≤ 0.05 according to Tukey’s pairwise comparison tests.

GA3 treatment caused a significant induction of the expression of five *SlGRF* genes, namely *SlGRF2*, *SlGRF3*, *SlGRF4*, *SlGRF5* and *SlGRF6* (Figure 6a). Relative to the control, the transcript levels of *SlGRF2* and *SlGRF3* were increased 1.5- to 3-fold at 9 and 24 h after treatment (*p* ≤ 0.05, Figure 6a). The transcript level of *SlGRF4* was upregulated approximately 2-fold at 3 h after treatment compared to control (*p* ≤ 0.05, Figure 6a). *SlGRF5* was gradually down-regulated from 3 to 24 h after treatment, whereas *SlGRF6* was upregulated only at 1 h after treatment but that was downregulated at 3 and 9 h after treatment (*p* ≤ 0.05, Figure 6a). The expression levels of the other six *SlGRF* genes were either only slightly induced or not affected by GA3 treatment (Figure 6a).

#### 2.7.2. Abscisic Acid Treatment

Following abscisic acid (ABA) treatment, the *SlGRF2*, *SlGRF3*, *SlGRF4*, *SlGRF5, SlGRF6, SlGRF7*, *SlGRF10, SlGRF12* and *SlGRF13* genes showed significant variation in expression at different time points compared to the control (Figure 6b). *SlGRF7* was downregulated at all four time-points compared to the control (*p* ≤ 0.05, Figure 6b). The expression levels of *SlGRF4* and *SlGRF12* were down-regulated from 1 to 24 h after treatment in comparison with the control (*p* ≤ 0.05, Figure 6b). *SlGRF10*, *SlGRF11* and *SlGRF13* were down-regulated at 9 and 24 h after treatment compared to control. *SlGRF2* and *SlGRF3* showed increasing expression only at 9 h after treatment compared to control.

#### 2.7.3. Jasmonic Acid Treatment

Most of the *SlGRF* genes were down-regulated at early stage of jasmonic acid (JA) treatment except *SlGRF5* and *SlGRF12* (*p* ≤ 0.01, Figure 6c). Both of those two genes were upregulated at 1 h of JA treatment compared to control. Retarded expression of all of *SlGRF* genes was observed at 9 h of JA treatment. By contrast, relatively higher level of expression was found in case of *SlGRF1*, *SlGRF6*, *SlGRF7*, and *SlGRF10* at later stage (24 h) of JA treatment although expression was not too much striking compared to control (Figure 6c).

## 3. Discussion

Recent studies showed the involvement of GRFs not only in leaf and stem development, but also in flowering, regulation of plant longevity, seed and root development and the control of growth under stress conditions [3,4,6,8,16,21,23,25]. However, it is still unknown whether *GRF* genes also affect growth, development and defense related processes in tomato. In this study, we reported 13 *GRF* genes in *Solanum lycopersicum*, whereas the *Arabidopsis* and rice genome contain nine and twelve *GRF* genes, respectively, [5,11].

In addition to the N-terminal conserved QLQ and WRC domains reported by Cao et al. [24], the SlGRF proteins further contain C-terminal amino acid motifs such as TQL, FFD similar to *Arabidopsis* GRF proteins [8]. These C-terminal motifs have significant role in the GRF proteins function in different plant tissues and organs [26]. The diverge C-terminal proteins of GRFs are also responsible for the transcriptional transactivation activity by acting as binding sites for other proteins such as transcriptional co-regulators in tomato [6]. Both N-terminal and C-terminal domains of tomato GRF proteins ultimately regulate plant growth and development [4,10,11]. Our phylogenetic analysis revealed the evolutionary relationships between the tomato GRF proteins and also showed that the tomato GRF proteins were more closely clustered with potato GRFs compared to other crop GRF proteins, indicating that they may have evolved from common ancestors. The absence of tomato, potato, and Arabidopsis GRF proteins belonging to subfamilies B and I indicated that these were either acquired in rice and maize lineages after divergence from the last common ancestor or lost in tomato, potato, and *Arabidopsis*.

A pseudogene is a non-functional copy of a gene that can be widely distributed in a eukaryotic genome by retro-transposition of messenger RNA (mRNA) or by duplication of genomic DNA. *SlGRF9* encodes a predicted protein much smaller than the other SlGRF proteins and its transcription was undetectable in the various tissues examined. This indicates that *SlGRF9* might be a pseudogene in the tomato genome. The presence of such predicted pseudogenes has previously been reported in *Brassica rapa* [12].

The functional diversity of genes can be predicted from their differential expression in different tissues. We therefore studied the expression patterns of *SlGRF* family genes in various tissues and found that most of the genes are highly expressed in flower buds compared with other organs (Figure 4). Flower bud initiation and development is of great importance in fruit set and cultivation. The transition phase from the vegetative state to the reproductive state, i.e., the induction and development of flower buds, is regulated by several floral genes and environmental and physiological factors [27]. The high expression of most *SlGRF* genes in flower bud suggests that they may function in flower bud formation and in flower development through the association with other flower-specific genes. The role of *GRF* genes in regulation of flower development has been reported in several recent studies [14,15,16]. *GRF* genes in rice are strongly expressed in immature leaves, flower buds and shoot tips and are involved in plant growth and development by regulating cell proliferation in actively growing tissue [10,11]. OsGRF1 controls flowering time in addition to regulating leaf growth in rice [28], and OsGRF6 is involved in floral organ development [4]. In addition to their high expression levels in flower buds, a comparatively higher expression of *SlGRF7*, *SlGRF10*, *SlGRF11*, *SlGRF12* and *SlGRF13* in the meristem, similar with their orthologs *Arabidopsis* (*AtGRF2*, *AtGRF3*, *AtGRF4*, and *AtGRF8*) counterparts indicating their possible involvement in meristem function and organ formation [5]. Higher expression of *SlGRF6* in roots compared to stem, leaf and developing fruits, like its putative orthologous of *AtGRF1* and *AtGRF2* suggests a similar role in this organ [5,8]. When compared with RNA-Seq data obtained from Solgenomics database, our expression data matched with RNA-Seq data for majority *SlGRF* genes with a few exceptions, supporting the reliability of our data (Figure S3).

Tomato is used as a model plant for studying the development and ripening of climacteric fruit [29]. Cell division, cell expansion and ripening are three critical stages of tomato fruit development [30]. Auxins, ethylene and gibberellins are hormones that play major roles in fruit development, possibly through the control of expression of genes that contain *cis*-elements in their promoter region [31,32,33]. In this study, the expression levels of *SlGRF* genes in developing fruit were very low compared with those in other vegetative and reproductive tissues (Figure 4). However, the comparatively high expression of *SlGRF2*, *SlGRF3*, *SlGRF5*, *SlGRF12* and *SlGRF13* in small green fruits and that of *SlGRF1* in ripening fruits (Figure 4) indicated their possible function in tomato fruit development. The tissue-specific or stress-responsive expression patterns of multi-stimulus-responsive genes are often determined by *cis*-regulatory elements [34]. We found that most of the *SlGRF* genes have one or more than one of the following phytohormone-responsive cis-elements in their promoter regions such as: auxin-, ethylene- and gibberellin-responsive cis-elements. In addition, other cis-elements (e.g., O2-site, GC-motif, as-2-box) present in 13 *SlGRF* genes are diverse in functions and often complementary among the *SlGRF* genes. Considering the diversity of function and distribution of *cis*-elements in the promoter regions of those genes, we speculate that they may differentially regulate the expression of genes that are involved in the development and ripening of tomato fruits. Therefore, further functional characterization of the *SlGRF* genes is highly important to attain new insights about the molecular mechanism of fruit development and ripening. It has been similarly suggested based on their expression profiles that *CsGRF* (*Citrus sinensis GRF)* genes are involved in cell expansion and the development of citrus fruit [35].

Plants have evolved sophisticated signaling and defense systems to withstand stress conditions. The activation of stress-responsive genes increases plant tolerance to overcome unfavorable circumstances [36,37]. AtGRF7 of *Arabidopsis* acts as a co-activator of Dehydration Responsive Element 2A (*DREB2A*) and other stress-responsive genes that eventually provide increased resistance to osmotic and drought stress, which is attributed to higher expression levels of stress-responsive genes under stress conditions [23]. GRF transcription factors have been reported to play important roles in plant growth through regulating defense signaling and stress responses [21,23,38,39,40,41]. For example, Arabidopsis Growth regulating Factor 1 and 3 (*AtGRF1* and *AtGRF3*) are reported to play a central role in the coordination of plant growth with defense signaling and stress responses [40]. In addition, Büyük and Aras [42] reported the correlation between *Phaseolus Vulgaris GRF* (*PhvGRF*s) genes and drought stress response in a cultivar-specific manner in common bean. In our study, variable expression of most of the *SlGRF* genes namely *SlGRF1*, *SlGRF2*, *SlGRF3*, *SlGRF4*, *SlGRF7* and *SlGRF10* (from >1-fold to >5-fold) under the exposure to NaCl, heat, cold and drought stresses suggested that several tomato *GRF* genes have similar biological functions in response to abiotic (NaCl, heat, cold, drought stress) stress as either positive or negative regulators. 

Phytohormones act as endogenous messengers and organize various signal transduction pathways allowing plants to respond against stresses [43,44,45], whereas GA plays an important role in plant growth and development, specifically in seed germination, stem and leaf elongation, flower induction and anther, fruit and seed development [46]. GA3 acts as a repressor of *Knotted1-like homeobox* genes, which inhibit cell differentiation in the shoot apical meristem (SAM) [47]. The GRFs act as positive regulators of gibberellin production and act as repressors of *KNOX* gene expression [20,48]. The expression levels of *SlGRF2*, *SlGRF3*, *SlGRF4*, *SlGRF5* and *SlGRF6* are regulated (increased or repressed) by GA3 treatment, suggesting that those *SlGRF*s might positively regulate the GA3 production. The other six genes were not significantly regulated by GA3 treatment, indicating that they might not be involved in GA response regulation in tomato similar to the *Arabidopsis* (e.g., *AtGRF1–AtGR9)* and rice (e.g., *OsGRF4*, *OsGRF5*, *OsGRF6*, *OsGR9*, and *OsGRF11) GRF* genes [8,11]. By contrast, in our analysis, several of the abiotic stress-induced genes, namely *SlGRF1*, *SlGRF4*, *SlGRF5*, *SlGRF7*, *SlGRF10*, *SlGRF11* and *SlGRF12*, were induced by exogenous ABA treatment and some others did not show any significant change after ABA treatment. These results suggest the involvement of *SlGRF* genes in abiotic stress tolerance via both ABA-dependent and ABA-independent signaling pathways since ABA plays a critical role in integrating various stress signals, such as salinity, drought and cold, and controlling downstream stress responses [43,48].

JA believed to play an important role in growth, development and various physiological processes in plant including storage organ formation, reproductive processes, fruit ripening, senescence and biotic and abiotic stress tolerance [49,50,51,52]. Exogenous application of JA so far tested to analyze their relatedness with *GRF* genes in improving crop yield and quality in different plants under stress or non-stress conditions. We found that the expression level of eleven *SlGRF* genes was weakly affected (≤1 fold) by exogenous JA treatment. Among them, expressions of *SlGRF1*, *SlGRF6*, *SlGRF7*, and *SlGRF10* genes were induced only at 24 h after treatment indicating their possible function at the later stage of treatment. The down-regulated expressions of *SlGRF11*, *SlGRF12*, and *SlGRF13* in all the time point after treatment indicate their anatagonistic function in response to JA treatment. 

Together with all phytohomone treatment data are provided evidence that different *SlGRF* genes might have variable roles in the specific responses to different phytohormone treatments. The key results of this study have been summarized for getting insight the findings at a glance (Table 2).

## 4. Materials and Methods

### 4.1. Identification of GRF Family Genes and GRF Proteins in Tomato

We retrieved 15 tomato *GRF* gene sequences from the Solgenomics database (https://solgenomics.net/) [53]. Among them, 13 encoded both the QLQ and WRC domains whereas the other two encoded only the WRC domain. We selected the 13 *GRF* genes with both domains for further analysis. We confirmed that the 13 *GRF* genes were present in tomato using SOL Genomics Network (SGN) (https://solgenomics.net/search/locus) [54]. iTAK-Plant Transcription factor and Protein Kinase Identifier and Classifier (http://bioinfo.bti.cornell.edu/cgi-bin/itak/index.cgi) [55], and Tomato genomic Resources database (http://59.163.192.91/tomato2/getTF_family.php?trans_fac_family=zf-HD) [56]. Conserved domains were identified using the SMART conserved domain search tool (http://smart.embl-heidelberg.de/) [57] and Pfam (http://www.sanger.ac.uk/science/tools/pfam) [58] databases. The NCBI (National Centre for Biotechnology Information) ORF (Open Reading Frame) finder tool (https://www.ncbi.nlm.nih.gov/orffinder/) [59] was used to determine the open reading frames of tomato *GRF* genes. The number, MW and pI of putative tomato GRF proteins were determined using the ProtParam tool (http://web.expasy.org/protparam/) [60] Phylogenetic analysis of GRF proteins from tomato and other plants was performed by the MEGA 6.0 software using the UPGMA (Unweighted Pair Group Method with Arithmetic Mean) tree with the following parameters: Poisson correction, pair-wise deletion and bootstrap values in percentages with 1000 replicates [61]. The Gene Structure display Server (GSDS) (http://gsds.cbi.pku.edu.cn/) [62] was used to analyze the exon–intron distribution of tomato *GRF* genes. A multiple protein sequence alignment was performed using the Genedoc (https://www.nrbsc.org/gfx/genedoc/ebinet.htm) [63] multiple sequence alignment tool following ClustalW parameters. Conserved motifs in the full-length protein sequences from tomato, *Arabidopsis* and rice were identified with the Multiple EM for Motif Elicitation (MEME) web tool (http://meme-suite.org/) [64]. The analysis conditions were set as follows: maximum number of motifs 10, minimum width 6, and maximum width 50.

### 4.2. Identification of Cis-Acting Elements and Chromosomal Position of Tomato GRF Genes

Putative *cis*-regulatory elements (approximately 5 to 10 bp) of 13 tomato *GRF* genes were identified by analyzing approximately 1500 bp upstream sequences from the ATG start codon of each gene using the PlantCARE web-based tool (http://bioinformatics.psb.ugent.be/webtools/plantcare/html/) [65]. The chromosomal positions of 13 tomato *GRF* genes were identified from the Sol Genomics database and their distribution on the chromosome was analyzed using the MapGene2Chrom web v2 software (http://mg2c.iask.in/) [66].

### 4.3. Functional Analysis of Tomato GRF Genes

The putative molecular and biological functions and cellular localization of the 13 tomato GRF proteins were assessed using the Blast2GO (https://www.blast2go.com/) [67] functional annotation and genomics software. The putative amino acid sequences were loaded in FASTA format into the Blast2GO program, and QBlast from NCBI was performed. Subsequently, mapping, annotation and interproscan of GO terms associated with each query, were carried out sequentially to predict protein function.

### 4.4. Plant Sample Collection

Tomato (*S lycopersicum* L. cv. Ailsa Craig) seeds were germinated in potted soil in a growth chamber and seedlings were maintained in a controlled environment at 25 °C day/20 °C night, a 16-h light/8-h dark photoperiod, a relative humidity ranging from 55% to 70%, and a light intensity of 300 μmol m^−2^ s^−1^. Roots, stems, leaves, seedlings and shoot meristem with 2–3 leaf primordia were collected using tweezers from four-week-old seedlings. For collection of other samples, the seedlings were transferred to a greenhouse to allow further growth. The culture condition of greenhouse was as follows: temperature 18 ± 2 °C, relative humidity between 65% and 80%. The plants were transferred to the greenhouse during spring season. The following samples were then collected: (i) flower bud; (ii) full blooming flowers at the anthesis stage; (ii) developing fruits of approximately 0.8–1.0 cm in diameter and approximately 14 days after pollination; (iii) developing fruits of approximately 2 cm in diameter and approximately 20 days after pollination; (iv) mature green fruits approximately 45 days after pollination; (v) fruits at breaker; (B) stage when the green color of the mature fruits changes to light yellow orange; (vi) fruits 3 days after the breaker stage (B3); and (vii) fruits 7 days after the breaker stage (B7). Three biological replicates for each condition were used, and each biological replicate was sampled three times. Seedlings of comparable growth at 28 days of age were sprayed with 100 µM GA3 and ABA for GA3 and ABA treatment, respectively. The seedlings were sprayed with 50 µM JA to impose JA treatment [54]. For cold and heat treatments, the seedlings were incubated at 4 °C and 40 °C in a growth cabinet, respectively for 24 h [54,55]. The seedlings were gently pulled from the soil and the root system was cleaned with fresh water. Seedlings were subsequently put on a dry paper towel to simulate drought (desiccation) condition for 24 h [68]. For NaCl treatment, seedlings were treated with a 200 mM NaCl solution to raise electrical conductivity level up to 15 dSm^−1^ at the rhizosphere [68,69]. All treatments were repeated 3 times. Plants growing in pots under normal conditions (25 °C) were sampled as control at 0 h after treatment for heat and cold stress. Although drought was applied by putting plants on paper towels, plants in potting soil under normal conditions were sampled as the control for drought treatment. Leaf samples from all stress- treated seedlings were collected at 0 (control), 1, 3, 9 and 24 h of treatment for expression analysis. All plant samples were frozen in liquid nitrogen and stored at −80 °C for further use.

### 4.5. RNA Extraction and cDNA Synthesis

Total RNA from different organs was extracted using the Qiagen RNeasy mini kit (QIAGEN, Hilden, Germany) according to the manufacturer’s protocol. The extracted RNA was purified (removing the genomic DNA contaminants) using the QIAGEN RNase free DNase1 kit. The amount of RNA (quantity/quality) was measured with a NanoDrop^®^ 1000 Spectrophotometer (Wilmington, DE, USA). One µg of total RNA was used to synthesize cDNA using the Superscript^®^ III First-Strand cDNA synthesis kit that uses oligo dT primer (Invitrogen, Carlsbad, CA, USA).

### 4.6. qRT-PCR Expression Analysis

Primer3 software (http://bioinfo.ut.ee/primer3-0.4.0/primer3/input.htm) [70] was used to design a specific primer for the 13 *SlGRF* genes (Table S1). We designed several primers for the *SlGRF9* gene in attempts to analyze its expression in different organs but it was undetectable in all tissues tested. It may be that it was not expressed; alternatively, it might have had spatial and temporal expression patterns that precluded its detection in the tissues and stages that we tested. Both forward and reverse primers were designed on exon region of the gene (Table S1). To check the primer specificity, the designed primers and the associated homologus genes found in solgenomics were aligned using ClustalW software. Further, a melting curve analysis was also performed to confirm the specificity of each primer set. Efficiency of each primer set was tested after running a dilution series following Robin et al. 2016 (Table S2) [71]. *EF1a* (F: 5’-TCAGGTAAGGAACTTGAGAAGGAGCCT-3’, R: 5’-AGTTCACTTCCCCTTCTTCTGGGCAG-3’) [72] was used as a control gene for normalization. The qRT-PCR expression analyses of the *SlGRF* genes were performed using the LightCycler96 (Roche, Mannheim, Germany) thermal cycler. A total volume of 10 µL reaction mixture, containing 1 µL of 50 ng cDNA, 2 µL forward and reverse primers of 10 pmol concentration, 5 µL iTaqTM SYBR^®^ Green PCR kit (PCRBIOSYSTEMS, London, UK) and 2 µL double distilled water was prepared to conduct qRT-PCR analysis. The qRT-PCR reaction conditions were set to: pre-denaturation at 95 °C for 300 s followed by 40 cycles at 94 °C for 10 s, annealing at 58 °C for 10 s and extension at 72 °C for 15 s. The melting temperature was set to 95 °C for 10 s, 65 °C for 60 s and 97 °C for 1 s. The relative expression levels of the tomato *SlGRF* genes were normalized against the house-keeping gene, and the relative amount of the amplified product was calculated following the 2^−∆∆Ct^ method using root sample as calibrator for the expression analysis in different organs and leaf samples collected at 0 h after treatment was the calibrator for abiotic stress and hormone treatments [73].

### 4.7. Statistical Analyses

Statistical significance of the differences in relative expression levels of each gene between treatments (control versus stress) and of the differences in expression levels between time points within a treatment was determined with one-way analysis of variance (ANOVA) using the MINITAB statistical software 17 (Minitab Inc., State College, Pennsylvania, PA, USA). The mean separation of expression values was analyzed using Tukey’s pairwise comparison test.

## 5. Conclusions

This study systematically characterized *SlGRF* family genes using different bioinformatics approaches and transcript expression analysis. We analyzed their intron–exon organizations, chromosomal distributions, gene structures, evolutionary relationships and expression profiles in different tissues and under different stress conditions to predict their possible biological functions. The *SlGRF* genes are variably expressed in different tissues and fruits at different developmental stages with particularly high expression in flower buds, and meristems. The increased expression of *SlGRF* genes in response to abiotic stress and phytohormone treatments implies their function in growth and development of tomato plants under different stress conditions. Together, our results obtained from gene structure, phylogenetic relationships and transcript expression profiles in different tissues and under different stresses facilitate the identification of tomato *GRF* genes that might play roles in specific developmental processes and/or environmental stress conditions.

## Figures and Tables

**Table 1:** Sequence characteristics of *SlGRF* (*Solanum lycopersicum* growth-regulating factor) genes and corresponding proteins.

Serial No.	Gene Name	Locus Name	ORF (bp)	Chrom. (strand)	No. of Introns	Protein				
						Length (aa)	Domain (Start–End)		MW (kDa)	pI
							QLQ	WRC		
1	*SlGRF1*	Solyc12g096070	1038	C12(+)	3	345	9–45	72–114	38.5907	7.13
2	*SlGRF2*	Solyc08g005430	1071	C08(+)	2	356	30–66	93–135	39.1162	8.63
3	*SlGRF3*	Solyc08g075950	1086	C08(−)	2	361	27–63	90–132	39.8591	8.43
4	*SlGRF4*	Solyc07g041640	1035	C07(−)	2	344	10–46	79–121	39.59	8.99
5	*SlGRF5*	Solyc04g077510	1788	C04(+)	3	595	136–172	205–247	64.1118	8.3
6	*SlGRF6*	Solyc02g092070	1704	C02(+)	4	568	139–175	210–252	61.3674	8.51
7	*SlGRF7*	Solyc08g083230	1197	C08(+)	3	398	74–108	125–167	43.7076	7.92
8	*SlGRF8*	Solyc03g082430	1377	C03(−)	3	458	58–94	117–159	50.4247	5.97
9	*SlGRF9*	Solyc08g068760	468	C08(+)	0	155	46–82	106–143	17.3958	8.84
10	*SlGRF10*	Solyc01g091540	1263	C01(+)	2	420	57–93	122–163 311–352	46.5821	9.18
11	*SlGRF11*	Solyc09g009200	1146	C09(−)	3	381	79–115	150–192	42.0369	9.08
12	*SlGRF12*	Solyc10g083510	1170	C10(−)	3	389	81–115	157–199	42.3790	9.18
13	*SlGRF13*	Solyc08g079800	657	C08(−)	3	218	49–83	112–154	24.7856	8.95

ORF: Open reading frame; bp: Base pair; Chrom.: Chromosome; aa: Amino acid; pI: Isoelectric point; MW: Molecular weight; kDa: Kilodalton.

**Table 2:** Summarization of key findings with specific features of tomato *GRF* genes for organ development, abiotic stresses response, and different phytohormones treatment with their structural characteristics and phylogenetic classification.

Observation on	Key Findings and Predicted Specific Function (s) Based on Expression Data	
Structural characteristics	13 *GRF* genes were identified from *Solanum lycopersicum*. All of them contained functional QLQ and WRC domain and diverge C-terminal region rich in Pro, Gln, Ser/Thr that are frequently found in transcription factors. Besides, some of SlGRF contained C-terminal FFD and TQL motifs. The structural characteristics suggested that SlGRF proteins function as transcriptional regulators.	
Phylogenetic classification	The GRF proteins from tomato, potato, *Arabidopsis*, Chinese cabbage, rice and maize phylogenitically classified into nine subfamilies which indicated their ancestral evolution and tomato GRF proteins are more closely related to potato suggested their evolution from common ancestor.	
Relative expression in different organs/tissues	Among the 13 *SlGRF* genes one gene; *SlGRF9* was undetectable in all organ studied and suggested as pseudogene. The organ expression analysis revealed that most of the genes predominantly expressed in flower bud indicating possible function in flower bud (i.e., reproductive organ development) in tomato. The *SlGRF* genes also showed differential expression in meristem, full blooming flower, leaf, stem, root, seedling and in different fruit developmental stages suggesting the important function in growth and development of tomato. The relatively higher expression of 12 *SlGRF* genes in different organs are listed below:	
	*SlGRF1:*	Flower bud, meristem and ripening fruit
	*SlGRF2:*	Flower bud, meristem, flower blooming and small green fruit
	*SlGRF3:*	Flower bud, meristem, flower blooming and small green fruit
	*SlGRF4:*	Flower bud
	*SlGRF5:*	Flower bud, meristem and small green fruit
	*SlGRF7:*	Flower bud and meristem
	*SlGRF8:*	Flower bud
	*SlGRF10:*	Flower bud, meristem and ripening fruit
	*SlGRF11:*	Flower bud and meristem
	*SlGRF12:*	Meristem, leaf, flower bud, small green fruit
	*SlGRF13:*	Meristem, leaf, flower bud, seedling, stem, and small green fruit
Relative expression under abiotic stresses and phytohormone treatments	Four abiotic stresses—NaCl, drought, heat, cold and three phytohormones (GA3, ABA, and JA) treatments—were studied where the following genes were (up/down) regulated by abiotic and phytohormone treatments at different time points:	
	*SlGRF1:*	NaCl, drought, heat, cold, JA
	*SlGRF2:*	NaCl, drought, heat, cold, ABA, GA3, JA
	*SlGRF3:*	NaCl, drought, heat, cold, ABA, GA3, JA
	*SlGRF4:*	NaCl, drought, heat, cold, ABA, GA3, JA
	*SlGRF5:*	NaCl, drought, heat, cold, ABA, GA3
	*SlGRF6:*	NaCl, drought, heat, cold, ABA, GA3, JA
	*SlGRF7:*	NaCl, drought, ABA, GA3, JA
	*SlGRF10:*	Drought, heat, cold, ABA, JA
	*SlGRF11:*	Drought, heat, cold, JA
	*SlGRF12:*	Drought, heat, cold, ABA, JA
	*SlGRF13:*	Drought, heat, cold, ABA, JA

## Supplementary Materials

Supplementary materials can be found at www.mdpi.com/1422-0067/18/5/1056/s1.

## References

[1] Kim J.H., Tsukaya H. (2015). Regulation of plant growth and development by the GROWTH-REGULATING FACTOR and GRF-INTERACTING FACTOR duo. J. Exp. Bot..

[2] Bao M., Bian H., Zha Y., Li F., Sun Y., Bai B., Chen Z., Wang J., Zhu M., Han N. (2014). miR396a-mediated basic helix-loophelix transcription factor *bHLH_74_* repression acts as a regulator for root growth in Arabidopsis seedlings. Plant Cell Physiol..

[3] Debernardi J.M., Mecchia M.A., Vercruyssen L., Smaczniak C., Kaufmann K., Inze D., Rodriguez R.E., Palatnik J.F. (2014). Post-transcriptional control of GRF transcription factors by microRNA miR396 and GIF co-activator affects leaf size and longevity. Plant J..

[4] Liu H., Guo S., Xu Y., Li C., Zhang Z., Zhang D., Xu S., Zhang C., Chong K. (2014). OsmiR396d-regulated OsGRFs function in floral organogenesis in rice through binding to their targets *OsJMJ706* and *OsCR4*. Plant Physiol..

[5] Omidbakhshfard M.A., Proost S., Fujikura U., Mueller-Roeber B. (2015). Growth-regulating factors (GRFs): A small transcription factor family with important functions in plant biology. Mol. Plant..

[6] Van der Knaap E., Kim J.H., Kende H. (2000). A novel gibberellins-induced gene from rice and its potential regulatory role in stem growth. Plant Physiol..

[7] Zhang D.F., Li B., Jia G.Q., Zhang T.F., Dai J.R., Li J.S., Wang S.C. (2008). Isolation and characterization of genes encoding GRF transcription factors and GIF transcriptional coactivators in Maize (*Zea mays* L.). Plant Sci..

[8] Kim J.H., Choi D., Kende H. (2003). The AtGRF family of putative transcription factors is involved in leaf and cotyledon growth in Arabidopsis. Plant J..

[9] Treich I., Cairns B.R., de los Santos T., Brewster E., Carlson M. (1995). SNF11, a new component of the yeast SNF-SWI complex that interacts with a conserved region of SNF2. Mol. Cell. Biol..

[10] Kim J.H., Kende H. (2004). A transcriptional coactivator, AtGIF1, is involved in regulated leaf growth and morphology in Arabidopsis. Proc. Natl. Acad. Sci. USA.

[11] Choi D., Kim J.H., Kende H. (2004). Whole genome analysis of the *OsGRF*gene family encoding plant-specific putative transcription activators in rice (*Oryza sativa* L.). Plant Cell Physiol..

[12] Wang F., Qiu N., Ding Q., Li J., Zhang Y., Li H., Gao J. (2014). Genome-wide identification and analysis of the growth-regulating factor family in Chinese cabbage (*Brassica rapa* L. ssp. pekinensis). BMC Genom..

[13] Kim H.J., Lee B.H. (2006). Growth-regulating factor 4 of *Arabidopsis thaliana* is required for development of leaves, cotyledons, and shoot apical meristem. J. Plant Biol..

[14] Yang F., Liang G., Liu D., Yu D. (2009). *Arabidopsis* miR396 mediates the development of leaves and flowers in transgenic tobacco. J. Plant Biol..

[15] Baucher M., Moussawi J., Vandeputte O.M., Monteyne D., Mol A., Pérez-Morga D., Jaziri M.E.I. (2013). A role for the miR396/GRF network in specification of organ type during flower development, as supported by ectopic expression of *Populus trichocarpa* miR396c in transgenic tobacco. Plant Biol..

[16] Liang G., He H., Li Y., Wang F., Yu D. (2014). Molecular mechanism of miR396 mediating pistil development in *Arabidopsis thaliana*. Plant Physiol..

[17] Liu J., Hua W., Yang H.L., Zhan G.M., Li R.J., Deng L.B., Wang X.F., Liu G.H., Wang H.Z. (2012). The *BnGRF2* gene (GRF2-like gene from *Brassica napus*) enhances seed oil production through regulating cell number and plant photosynthesis. J. Exp. Bot..

[18] Wynn A.N., Rueschhoff E.E., Franks R.G. (2011). Transcriptomic characterization of a synergistic genetic interaction during carpel margin meristem development in *Arabidopsis thaliana*. PLoS ONE.

[19] Ye R., Yao Q.H., Xu Z.H., Xue H.W. (2004). Development of an efficient method for the isolation of factors involved in gene transcription during rice embryo development. Plant J..

[20] Kuijt S.J., Greco R., Agalou A., Shao J., CJ’t Hoen C.O., Verna S.E., Osnato M., Curiale S., Meynard D., van Gulik R. (2014). Interaction between the growth-regulating factor and knotted1-like homeobox families of transcription factors. Plant Physiol..

[21] Hewezi T., Maier T.R., Nettleton D., Baum T.J. (2012). The Arabidopsis microRNA396-*GRF1/GRF3* regulatory module acts as a developmental regulator in the reprogramming of root cells during cyst nematode infection. Plant Physiol..

[22] Li D., Fu F., Zhang H., Song F. (2015). Genome-wide systematic characterization of the bZIP transcriptional factor family in tomato (*Solanum lycopersicum* L.). BMC Genom..

[23] Kim J.S., Mizoi J., Kidokoro S., Maruyama K., Nakajima J., Nakashima K., Mitsuda N., Takiguchi Y., ohmeoTakagi M., Kondou Y. (2012). Arabidopsis GROWTH-REGULATING FACTOR7 functions as a transcriptional repressor of abscisic acid and osmotic stress–responsive genes, including DREB2A. Plant Cell..

[24] Cao D., Wang J., Ju Z., Liu Q., Li S., Tian H., Fu D., Zhu H., Luo Y., Zhu B. (2016). Regulations on growth and development in tomato cotyledon, flower and fruit via destruction of miR396 with short tandem target mimic. Plant Sci..

[25] Horiguchi G., Kim G.T., Tsukaya H. (2005). The transcription factor AtGRF5 and the transcription coactivator AN3 regulate cell proliferation in leaf primordia of *Arabidopsis thaliana*. Plant J..

[26] Ahmadi J., Noormohammadi N., Ourang S.F. (2014). Identification of conserved domains and motifs for GRF gene family. Genome.

[27] Koutinas N., Pepelyankov G., Lichev V. (2010). Flower induction and flower bud development in apple and sweet cherry. Biotechnol. Biotechnol. Equip..

[28] Luo A.D., Liu l., Tang Z.S., Bai X.Q., Cao S.Y., Chu C.C. (2005). Down-regulation of *OsGrF1* gene in rice rhd1 mutant results in reduced heading date. J. Integr. Plant Biol..

[29] Karlova R., Chapman N., David K., Angenent G.C., Seymour G.B., de Maagd R.A. (2014). Transcriptional control of fleshy fruit development and ripening. J. Exp. Bot..

[30] Pesaresi P., Mizzotti C., Colombo M., Masiero S. (2014). Genetic regulation and structural changes during tomato fruit development and ripening. Front. Plant Sci..

[31] De Jong M., Mariani C., Vriezen W.H. (2009). The role of auxin and gibberellin in tomato fruit set. J. Exp. Bot..

[32] Le D.T., Nishiyama R., Watanabe Y., Vankova R., Tanaka M., Seki M., Tran L.S.P. (2012). Identification and expression analysis of cytokinin metabolic genes in soybean under normal and drought conditions in relation to cytokinin levels. PLoS ONE.

[33] Walther D., Brunnemann R., Selbig J. (2007). The regulatory code for transcriptional response diversity and its relation to genome structural properties in *A. thaliana*. PLoS Genet..

[34] Saeed A.I., Bhagabati N.K., Braisted J.C., Liang W., Sharov V., Howe E.A., Quackenbush J. (2006). TM4 Microarray software suite. Method Enzymol..

[35] Liu X., Guo L.X., Jin L.F., Liu Y.Z., Liu T., Fan Y.H., Peng S.A. (2016). Identification and transcript profiles of citrus growth-regulating factor genes involved in the regulation of leaf and fruit development. Mol. Biol. Rep..

[36] Heidel A.J., Clarke J.D., Antonovics J., Dong X. (2004). Fitness costs of mutations affecting the systemic acquired resistance pathway in Arabidopsis thaliana. Genetics.

[37] Sakuma Y., Maruyama K., Osakabe Y., Qin F., Seki M., Shinozaki K., Yamaguchi-Shinozaki K. (2006). Functional analysis of an *Arabidopsis* transcription factor, DREB_2_A, involved in drought-responsive gene expression. Plant Cell..

[38] Liu H., Tian X., Li Y., Wu C., Zheng C. (2008). Microarray-based analysis of stress-regulated microRNAs in *Arabidopsis thaliana*. RNA.

[39] Casadevall R., Rodriguez R., Debernardi J., Palatnik J., Casati P. (2013). Repression of growth regulating factors by the microRNA396 inhibits cell proliferation by UV-B radiation in *Arabidopsis* leaves. Plant Cell..

[40] Casati P. (2013). Analysis of UV-B regulated miRNAs and their targets in maize leaves. Plant Signal. Behav..

[41] Liu J., Rice J.H., Chen N., Baum T.J., Hewezi T. (2014). Synchronization of developmental processes and defense signaling by growth regulating transcription factors. PLoS ONE.

[42] Büyük İ., Aras S. (2016). Genome-wide in silico identification, characterization and transcriptional analysis of the family of growth-regulating factors in common bean (*Phaseolus vulgaris* L.) subjected to PEG-induced drought stress. Arch. Biol. Sci..

[43] Wolters H., Jurgens G. (2009). Survival of the flexible: Hormonal growth control and adaptation in plant development. Nat. Rev. Genet..

[44] Huang D., Wu W., Abrams S.R., Cutler A.J. (2008). The relationship of drought-related gene expression in *Arabidopsis thaliana* to hormonal and environmental factors. J. Expt. Bot..

[45] Fujita M., Fujita Y., Noutoshi Y., Takahashi F., Narusaka Y., Yamaguchi-Shinozaki K., Shinozaki K. (2006). Crosstalk between abiotic and biotic stress responses: A current view from the points of convergence in the stress signaling networks. Curr. Opin. Plant Biol..

[46] Singh D.P., Jermakow A.M., Swain S.M. (2002). Gibberellins are required for seed development and pollen tube growth in Arabidopsis. Plant Cell..

[47] Tuteja N. (2007). Abscisic acid and abiotic stress signaling. Plant Signal. Behav..

[48] Sakamoto T., Kamiya N., Ueguchi-Tanaka M., Iwahori S., Matsuoka M. (2001). KNOX homeodomain protein directly suppresses the expression of a gibberellin biosynthetic gene in the tobacco shoot apical meristem. Genes Dev..

[49] Browse J. (2009). Jasmonate: Preventing the maize tassel from getting in touch with his feminine side. Sci. Signal..

[50] Moreno J.E., Tao Y., Chory J., Ballare C.L. (2009). Ecological modulation of plant defense via phytochrome control of jasmonates sensitivity. Proc. Natl. Acad. Sci. USA.

[51] Nafie E., Hathout T., Al S., Al M. (2011). Jasmonic acid elicits oxidative defense and detoxification systems in *Cucumis melo* L. cells. Braz. J. Plant Physiol..

[52] Cipollini D. (2010). Constitutive expression of methyl jasmonates inducible responses delays reproduction and constrains fitness responses to nutrients in *Arabidopsis thaliana*. Evol. Ecol..

[53] Solgenomics database. https://solgenomics.net/.

[54] Locus in Solgenomics database. https://solgenomics.net/search/locus.

[55] iTAK-Plant Transcription factor and Protein Kinase Identifier and Classifier. http://bioinfo.bti.cornell.edu/cgi-bin/itak/index.cgi.

[56] Tomato genomic Resources database. http://59.163.192.91/tomato2/getTF_family.php?trans_fac_family=zf-HD.

[57] SMART conserved domain search tool. http://smart.embl-heidelberg.de/.

[58] Pfam database. http://www.sanger.ac.uk/science/tools/pfam.

[59] NCBI database. https://www.ncbi.nlm.nih.gov/orffinder/.

[60] ProtParam tool. http://web.expasy.org/protparam/.

[61] Tamura K., Stecher G., Peterson D., Filipski A., Kumar S. (2013). MEGA6: Molecular evolutionary genetics analysis version 6.0. Mol. Biol. Evol..

[62] The Gene Structure display Server. http://gsds.cbi.pku.edu.cn/.

[63] Genedoc multiple sequence alignment tool. https://www.nrbsc.org/gfx/genedoc/ebinet.htm.

[64] Multiple EM for Motif Elicitation (MEME) web tool. http://meme-suite.org/.

[65] PlantCARE database. http://bioinformatics.psb.ugent.be/webtools/plantcare/html/.

[66] MapGene2Chrom web v2 software. http://mg2c.iask.in/).

[67] Blast2GO functional annotation and genomics software. https://www.blast2go.com/.

[68] Khatun K., Robin A.H.K., Park J.I., Kim C.K., Kim K.B., Lim M.B., Lee D.J., Nou I.S., Chung M.Y. (2016). Genome-wide identification, characterization and expression *profiling of **ADF*** family genes **in**
*Solanum lycopersicum* L.. Genes.

[69] Khatun K., Robin A.H.K., Park J.I., Ahmed N.U., Kim C.K., Lim K.B., Kim M.B., Lee D.J., Nou I.S., Chung M.Y. (2016). Genome-wide identification, characterization and expression profiling of *LIM* family genes in *Solanum lycopersicum* L.. Plant Physiol. Biochem..

[70] Primer3 software. http://bioinfo.ut.ee/primer3-0.4.0/primer3/input.htm.

[71] Robin A.H.K., Yi G.E., Laila R., Yang K., Park J.I., Kim H.R., Nou I.S. (2016). Expression profiling of glucosinolate biosynthetic genes in *Brassica oleracea* L. var. *capitata* inbred lines reveals their association with glucosinolate content. Molecules.

[72] Aoki K., Yano K., Suzuki A., Kawamura S., Sakurai N., Suda K., Ooga K. (2010). Large-scale analysis of full length cDNAs from the tomato (*Solanum lycopersicum*) cultivar Micro-Tom, a reference system for the Solanaceae genomics. BMC Genom..

[73] Schmittgen T.D., Livak K.J. (2008). Analyzing real-time PCR data by the comparative CT method. Nat. Protoc..

