# Supplementary material for: Molecular Characterization and Expression Profiling of Tomato GRF Transcription Factor Family Genes in Response to Abiotic Stresses and Phytohormones
Source: Int J Mol Sci. 2017 May 13;18(5):1056. doi: 10.3390/ijms18051056 (PMC5454968; doi:10.3390/ijms18051056)
Supplement: Supplementary file 1 [file ijms-18-01056-s001.zip › ijms-189059_2proofreading_supplementary/Additional file 2.pptx]

## Slide 1
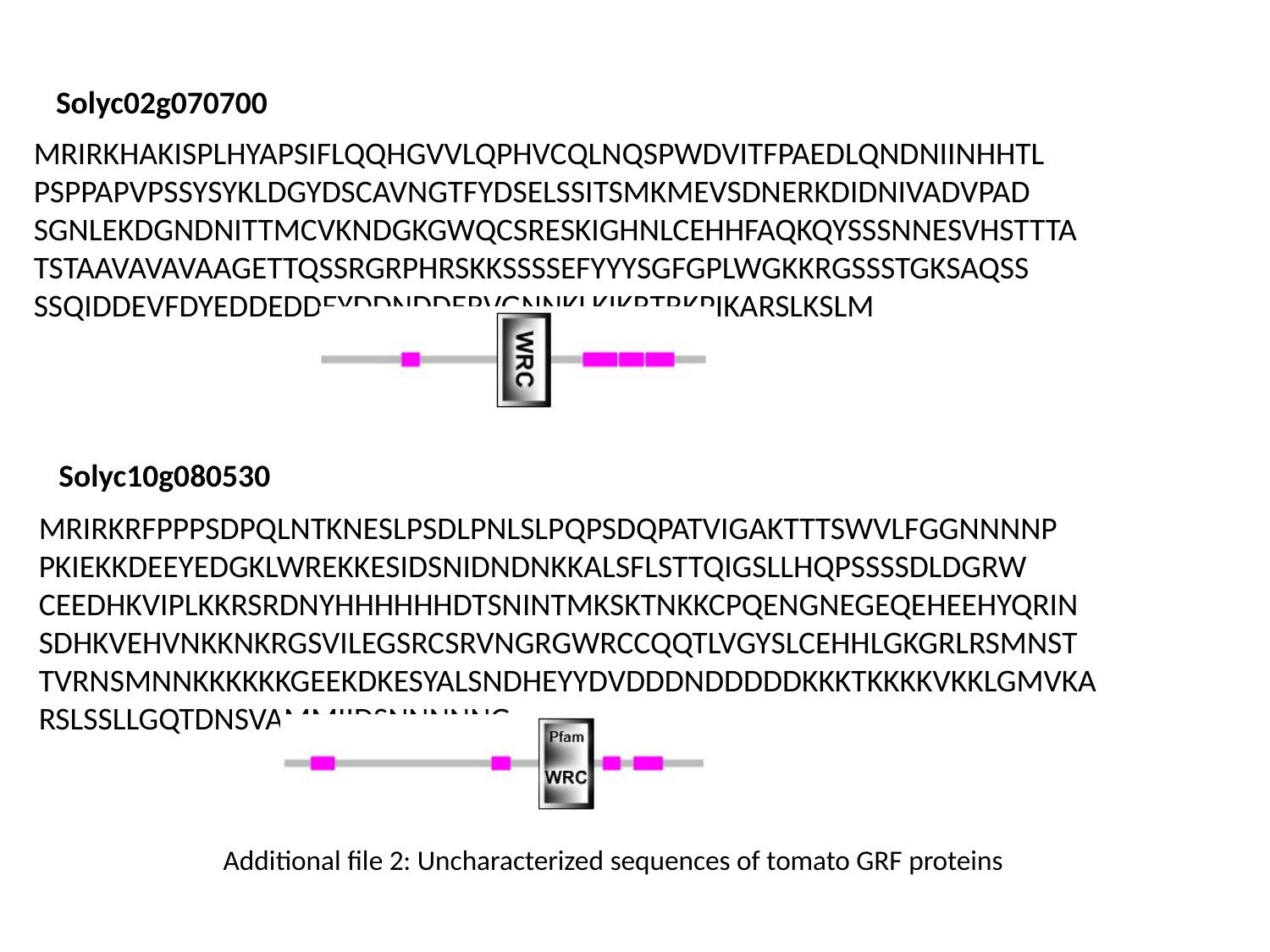

Solyc02g070700‍
MRIRKHAKISPLHYAPSIFLQQHGVVLQPHVCQLNQSPWDVITFPAEDLQNDNIINHHTL‍PSPPAPVPSSYSYKLDGYDSCAVNGTFYDSELSSITSMKMEVSDNERKDIDNIVADVPAD‍SGNLEKDGNDNITTMCVKNDGKGWQCSRESKIGHNLCEHHFAQKQYSSSNNESVHSTTTA‍TSTAAVAVAVAAGETTQSSRGRPHRSKKSSSSEFYYYSGFGPLWGKKRGSSSTGKSAQSS‍SSQIDDEVFDYEDDEDDEYDDNDDERVGNNKLKIKRTRKPIKARSLKSLM
Solyc10g080530
MRIRKRFPPPSDPQLNTKNESLPSDLPNLSLPQPSDQPATVIGAKTTTSWVLFGGNNNNP‍PKIEKKDEEYEDGKLWREKKESIDSNIDNDNKKALSFLSTTQIGSLLHQPSSSSDLDGRW‍CEEDHKVIPLKKRSRDNYHHHHHHDTSNINTMKSKTNKKCPQENGNEGEQEHEEHYQRIN‍SDHKVEHVNKKNKRGSVILEGSRCSRVNGRGWRCCQQTLVGYSLCEHHLGKGRLRSMNST‍TVRNSMNNKKKKKKGEEKDKESYALSNDHEYYDVDDDNDDDDDKKKTKKKKVKKLGMVKA‍RSLSSLLGQTDNSVAMMIIDSNNNNNG
Additional file 2: Uncharacterized sequences of tomato GRF proteins
