# Supplementary material for: Molecular Characterization and Expression Profiling of Tomato GRF Transcription Factor Family Genes in Response to Abiotic Stresses and Phytohormones
Source: Int J Mol Sci. 2017 May 13;18(5):1056. doi: 10.3390/ijms18051056 (PMC5454968; doi:10.3390/ijms18051056)
Supplement: Supplementary file 1 [file ijms-18-01056-s001.zip › ijms-189059_2proofreading_supplementary/ijms-189059_2proofreading_supplementary.docx]

Supplementary Materials: Molecular Characterization and Expression Profiling of Tomato GRF Transcription Factor Family Genes in Response to Abiotic Stresses and Phytohormones

Khadiza Khatun, Arif Hasan Khan Robin, Jong-In Park, Ujjal Kumar Nath, Chang Kil Kim, Ki-Byung Lim, Ill Sup Nou and Mi-Young Chung


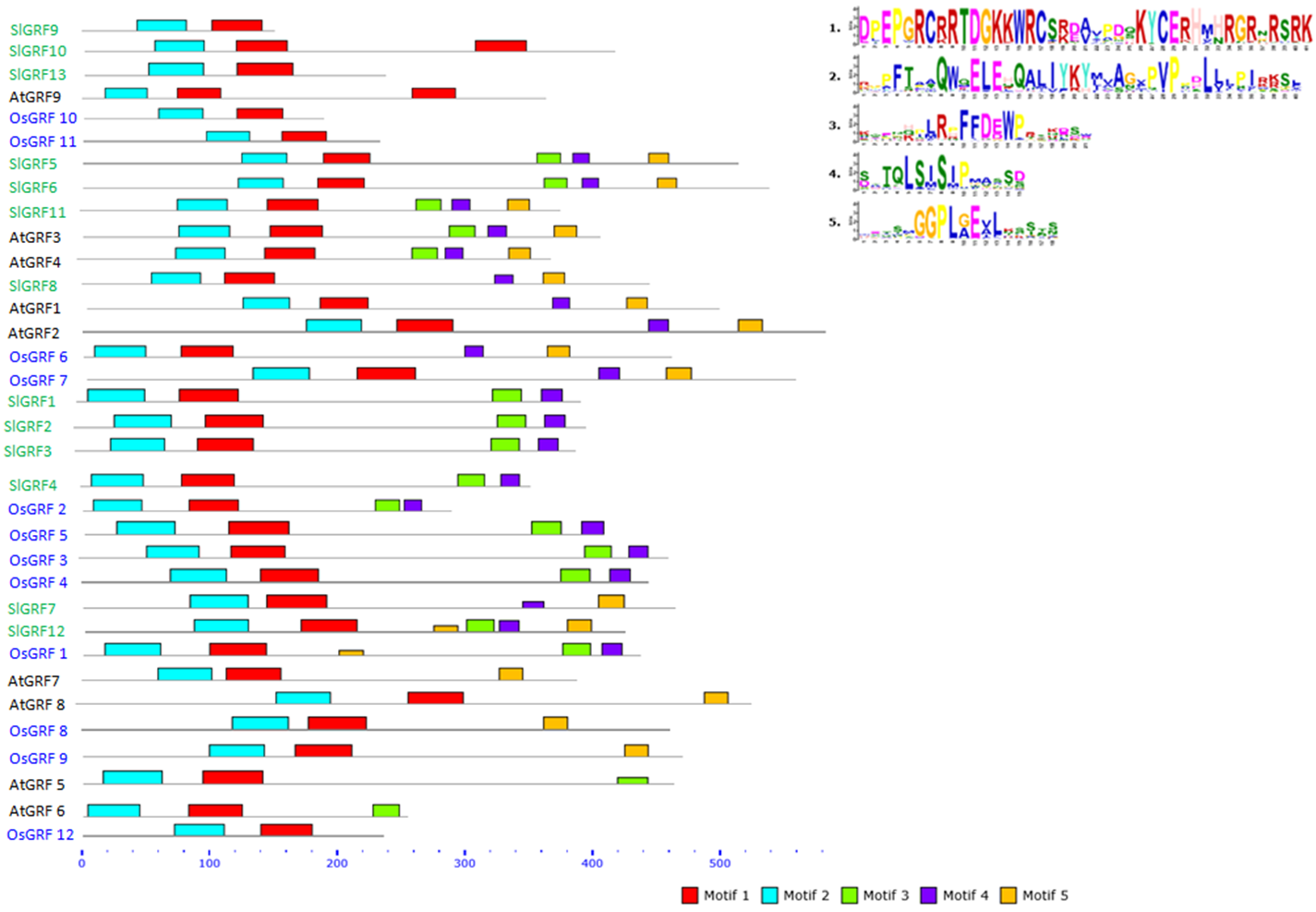


**Figure S1.** Conserved motif distribution in tomato, *Arabidopsis* and rice GRF proteins. The name of each member is shown on the left and different colors represent different motifs. The logos represent the predicted conserved motif sequences on the GRF protein sequences.

**
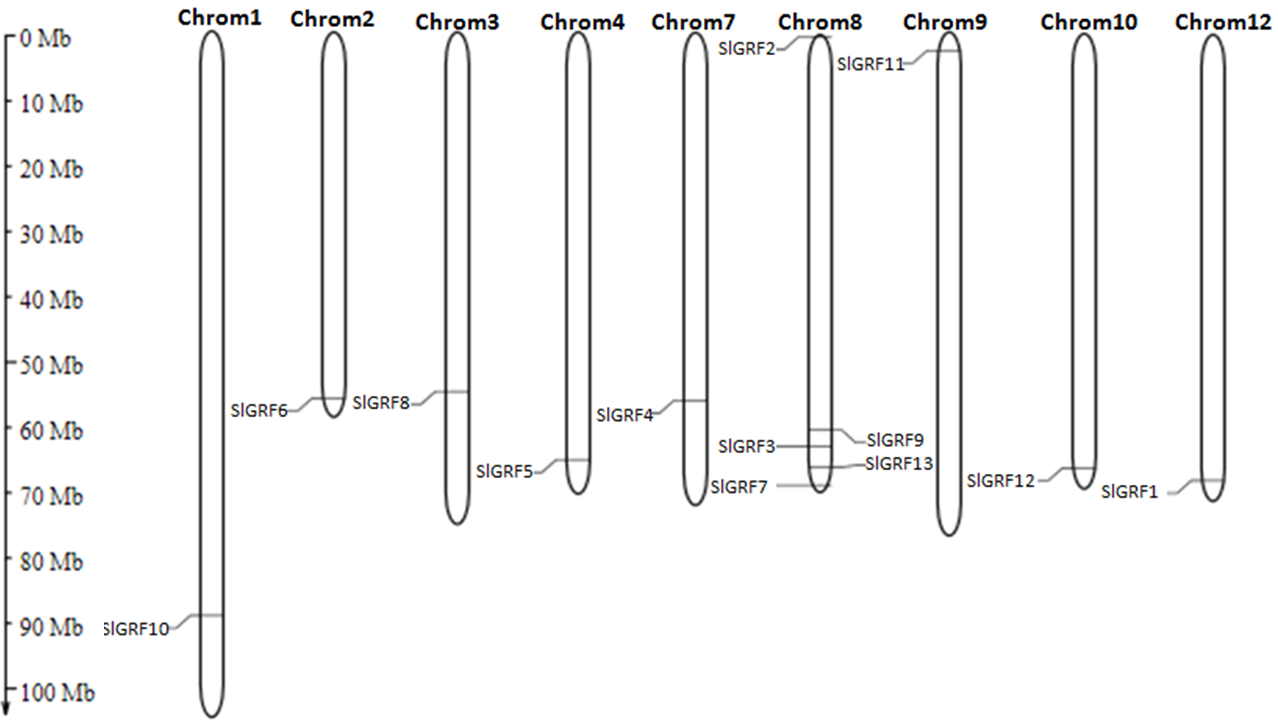
**

**Figure S2.** Distribution of *SlGRF* genes on tomato chromosomes. The chromosome (Chrom) number is indicated at the top of each chromosome representation.


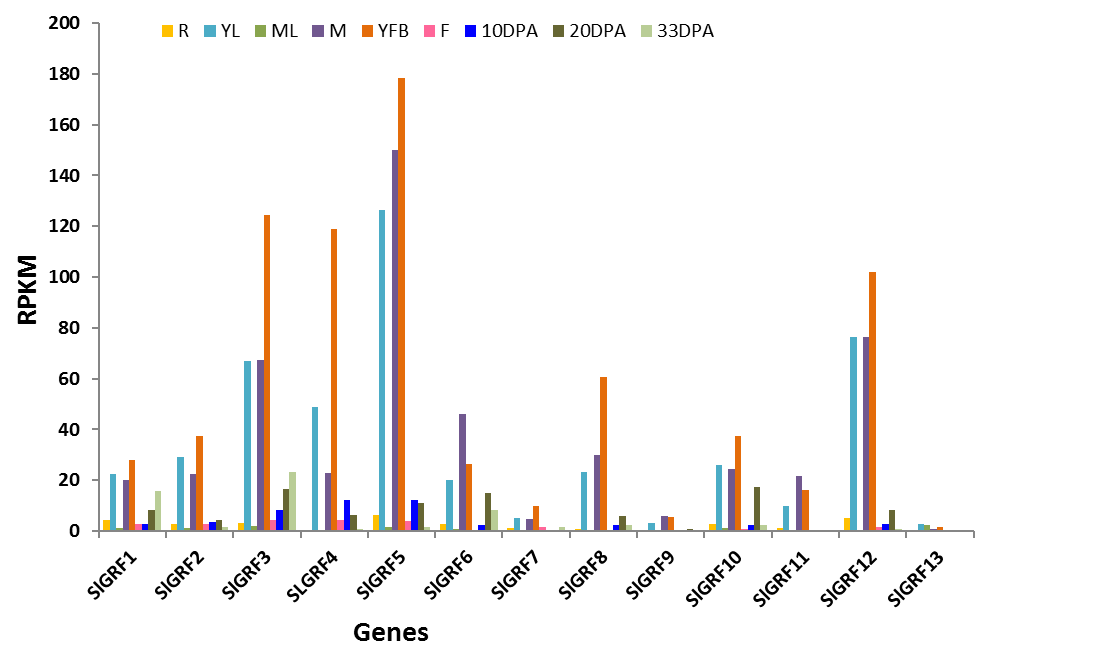


**Figure S3.** Putative expression profile of 13 *SlGRF* genes based on online RNA sequencing data downloaded from Solgenomics database (<https://solgenomics.net/>).

**Table S1.** Primer sequences used for qRT-PCR analysis of *SlGRF* genes.

**Table S2.** Ct values of *EF1a* and 12 *SlGRF* genes at five different concentrations (ng/µl) of cDNA representing the amplication efficiencies of primers used in qRT-PCR. Each data point represents an average of three qPCR observations.

| **cDNA concentration** | ***EF1a*** | ***SlGRF1*** | ***SlGRF2*** | ***SlGRF3*** | ***SlGRF4*** | ***SlGRF5*** | ***SlGRF6*** | ***SlGRF7*** | ***SlGRF8*** | ***SlGRF10*** | ***SlGRF11*** | ***SlGRF12*** | ***SlGRF13*** |
| --- | --- | --- | --- | --- | --- | --- | --- | --- | --- | --- | --- | --- | --- |
| 1  0.1  0.01  0.001  0.0001 | 16.77  20.07  23.37  26.67  30.3 | 25.9  29.1  32.4  35.9  39.4 | 24.96  28.3  31.2  34.5  38.5 | 25.5  29.1  32.4  35.7  39.2 | 26.09  31  34.3  37.6  40.9 | 25.2  28.5  31.8  35.1  38.7 | 27.9  31.3  34.6  37.9  41.2 | 25.4  28.7  32.1  35.4  38.7 | 25.9  29.5  32.8  36.1  40.1 | 25.8  29.1  32.4  35.7  39.2 | 27.9  30.9  34.7  38.5  40.9 | 26.7  30.5  33.8  37.1  40.4 | 27.3  30.9  34.9  38.2  40.6 |
| Primer efficiency values (%) | 98 | 98 | 100 | 97 | 89 | 99 | 100 | 100 | 93 | 99 | 98 | 97 | 97 |

**Table S3.** Sequence identity among the 13 GRF proteins of tomato.

Please define lettering in table footer.

**Table S4.** Putative *cis*-elements of more than 5 bp identified in 13 *GRF* genes from *Solanum lycopersicum* using PlantCARE database (URL).

| **Protein** | **A** | **B** | **C** | **D** | **E** | **F** | **G** | **H** | **I** | **J** | **K** | **L** | **M** |
| --- | --- | --- | --- | --- | --- | --- | --- | --- | --- | --- | --- | --- | --- |
| SlGRF1 (A) | 100 |  |  |  |  |  |  |  |  |  |  |  |  |
| SlGRF2 (B) | 73 | 100 |  |  |  |  |  |  |  |  |  |  |  |
| SlGRF3 (C) | 76 | 76 | 100 |  |  |  |  |  |  |  |  |  |  |
| SlGRF4 (D) | 38 | 39 | 39 | 100 |  |  |  |  |  |  |  |  |  |
| SlGRF5 (E) | 31 | 35 | 32 | 35 | 100 |  |  |  |  |  |  |  |  |
| SlGRF6 (F) | 33 | 33 | 50 | 56 | 41 | 100 |  |  |  |  |  |  |  |
| SlGRF7 (G) | 47 | 47 | 45 | 46 | 48 | 42 | 100 |  |  |  |  |  |  |
| SlGRF8 (H) | 53 | 58 | 56 | 47 | 43 | 46 | 50 | 100 |  |  |  |  |  |
| SlGRF9 (I) | 48 | 50 | 50 | 47 | 40 | 42 | 44 | 86 | 100 |  |  |  |  |
| SlGRF10 (J) | 53 | 52 | 55 | 49 | 43 | 37 | 42 | 48 | 46 | 100 |  |  |  |
| SlGRF11 (K) | 52 | 54 | 48 | 58 | 44 | 50 | 44 | 50 | 47 | 53 | 100 |  |  |
| SlGRF12 (L) | 41 | 45 | 49 | 40 | 42 | 52 | 29 | 43 | 37 | 49 | 57 | 100 |  |
| SlGRF13 (M) | 54 | 44 | 43 | 44 | 38 | 48 | 39 | 41 | 42 | 46 | 49 | 45 | 100 |

**Table S5.** Putative functions and cellular localizations of tomato GRF proteins.

| **Gene name** | **GO: Molecular function** | **GO: Biological process** | **GO: Cellular component** |
| --- | --- | --- | --- |
| *SlGRF1*  *SlGRF2*  *SlGRF3*  *SlGRF4*  *SlGRF5*  *SlGRF6*  *SlGRF7*  *SlGRF8*  *SlGRF9*  *SlGRF10*  *SlGRF11*  *SlGRF12*  *SlGRF13* | ATP binding; adenyl ribonucleotide binding, purine ribonucleoside binding, purine ribonucleotide binding, ribonucleotide binding, carbohydrate derivative binding, nucleoside phosphate binding, nucleoside binding, ion and anion binding  ATP binding; adenyl ribonucleotide binding, purine ribonucleoside binding, purine ribonucleotide binding, ribonucleotide binding, carbohydrate derivative binding, nucleoside phosphate binding, nucleoside binding, ion and anion binding  ATP binding; adenyl ribonucleotide binding, purine ribonucleoside binding, purine ribonucleotide binding, ribonucleotide binding, carbohydrate derivative binding, nucleoside phosphate binding, nucleoside binding, ion and anion binding  ATP binding; adenyl ribonucleotide binding, purine ribonucleoside binding, purine ribonucleotide binding, ribonucleotide binding, carbohydrate derivative binding, nucleoside phosphate binding, nucleoside binding, ion and anion binding  ATP binding; adenyl ribonucleotide binding, purine ribonucleoside binding, purine ribonucleotide binding, ribonucleotide binding, carbohydrate derivative binding, nucleoside phosphate binding, nucleoside binding, ion and anion binding  ATP binding; adenyl ribonucleotide binding, purine ribonucleoside binding, purine ribonucleotide binding, ribonucleotide binding, carbohydrate derivative binding, nucleoside phosphate binding, nucleoside binding, ion and anion binding  ATP binding; adenyl ribonucleotide binding, purine ribonucleoside binding, purine ribonucleotide binding, ribonucleotide binding, carbohydrate derivative binding, nucleoside phosphate binding, nucleoside binding, ion and anion binding  ATP binding; adenyl ribonucleotide binding, purine ribonucleoside binding, purine ribonucleotide binding, ribonucleotide binding, carbohydrate derivative binding, nucleoside phosphate binding, nucleoside binding, ion and anion binding  ATP binding; adenyl ribonucleotide binding, purine ribonucleoside binding, purine ribonucleotide binding, ribonucleotide binding, carbohydrate derivative binding, nucleoside phosphate binding, nucleoside binding, ion and anion binding  ATP binding; adenyl ribonucleotide binding, purine ribonucleoside binding, purine ribonucleotide binding, ribonucleotide binding, carbohydrate derivative binding, nucleoside phosphate binding, nucleoside binding, ion and anion binding  ATP binding; adenyl ribonucleotide binding, purine ribonucleoside binding, purine ribonucleotide binding, ribonucleotide binding, carbohydrate derivative binding, nucleoside phosphate binding, nucleoside binding, ion and anion binding  ATP binding; adenyl ribonucleotide binding, purine ribonucleoside binding, purine ribonucleotide binding, ribonucleotide binding, carbohydrate derivative binding, nucleoside phosphate binding, nucleoside binding, ion and anion binding  ATP binding; adenyl ribonucleotide binding, purine ribonucleoside binding, purine ribonucleotide binding, ribonucleotide binding, carbohydrate derivative binding, nucleoside phosphate binding, nucleoside binding, ion and anion binding | Regulation of transcription  DNA templated  Regulation of transcription  Regulation of transcription  Regulation of transcription  Regulation of transcription  Regulation of transcription  Regulation of transcription  Regulation of transcription  Regulation of transcription  Regulation of transcription  Regulation of transcription  Regulation of transcription  Regulation of transcription | Nucleus  Nucleus  Nucleus  Nucleus  Nucleus  Nucleus  Nucleus  Nucleus  Nucleus  Nucleus  Nucleus  Nucleus  Nucleus |

GO: Define.
