# Supplementary material for: Molecular Characterization and Expression Profiling of Tomato GRF Transcription Factor Family Genes in Response to Abiotic Stresses and Phytohormones
Source: Int J Mol Sci. 2017 May 13;18(5):1056. doi: 10.3390/ijms18051056 (PMC5454968; doi:10.3390/ijms18051056)
Supplement: Supplementary file 1 [file ijms-18-01056-s001.zip › ijms-189059_2proofreading_supplementary/ijms-189059_2proofreading_supplementary.pdf]

Supplementary Materials: Molecular Characterization and Expression Profiling of Tomato GRF Transcription Factor Family Genes in Response to Abiotic Stresses and Phytohormones

Khadiza Khatun, Arif Hasan Khan Robin, Jong-In Park, Ujjal Kumar Nath, Chang Kil Kim, Ki-Byung Lim, Ill Sup Nou and Mi-Young Chung

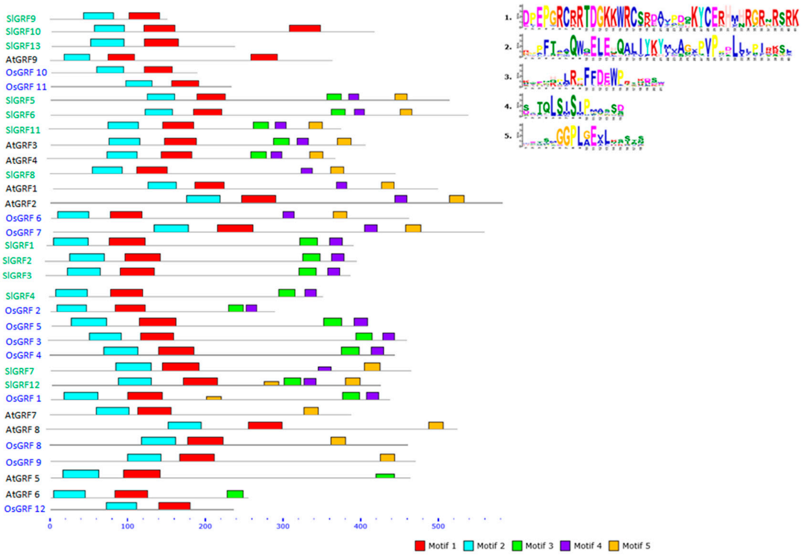

Figure S1. Conserved motif distribution in tomato, *Arabidopsis* and rice GRF proteins. The name of each member is shown on the left and different colors represent different motifs. The logos represent the predicted conserved motif sequences on the GRF protein sequences.

Formatted: Font: Italic

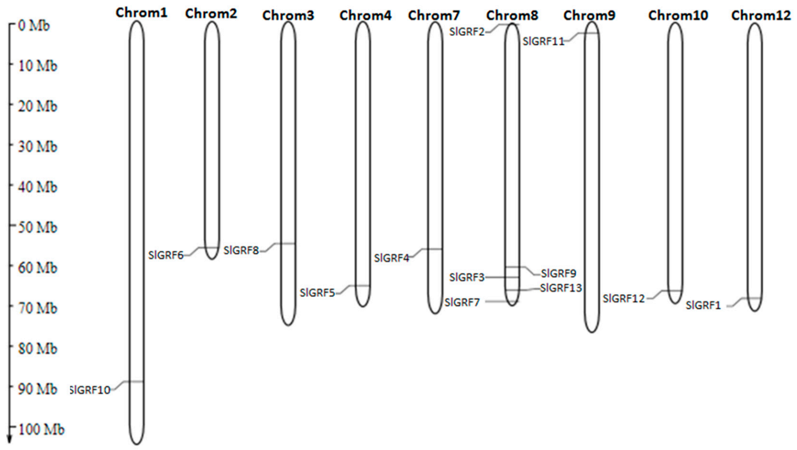

Figure S2. Distribution of *SlGRF* genes on tomato chromosomes. The chromosome (Chrom) number is indicated at the top of each chromosome representation.

Commented [l1]: Please provide a higher resolution image. Please note that "SlGRF10" on the left margin is incomplete, please revise.

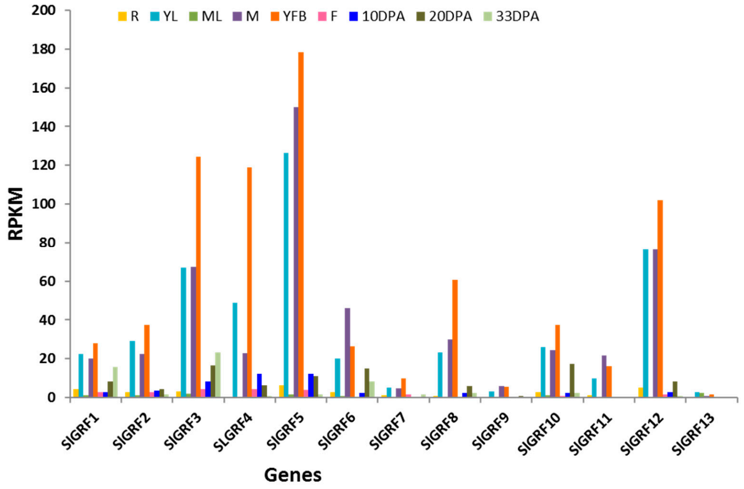

Figure S3. Putative expression profile of 13 *SlGRF* genes based on online RNA sequencing data downloaded from Solgenomics database (https://solgenomics.net/).

Commented [TW2]:

Table S1. Primer sequences used for qRT-PCR analysis of *SlGRF* genes.

Table S2. Ct values of *EF1a* and ~~twelve~~ 12 *SlGRF* genes at five different concentrations (ng/µl) of cDNA representing the amplication efficiencies of primers used in qRT-PCR. Each data point represents an average of three qPCR observations.

cDNA concentration	*EF1a*	*SlGRF1*	*SlGRF2*	*SlGRF3*	*SlGRF4*	*SlGRF5*	*SlGRF6*	*SlGRF7*	*SlGRF8*	*SlGRF10*	*SlGRF11*	*SlGRF12*	*SlGRF13*
1	16.77	25.9	24.96	25.5	26.09	25.2	27.9	25.4	25.9	25.8	27.9	26.7	27.3
0.1	20.07	29.1	28.3	29.1	31	28.5	31.3	28.7	29.5	29.1	30.9	30.5	30.9
0.01	23.37	32.4	31.2	32.4	34.3	31.8	34.6	32.1	32.8	32.4	34.7	33.8	34.9
0.001	26.67	35.9	34.5	35.7	37.6	35.1	37.9	35.4	36.1	35.7	38.5	37.1	38.2
0.0001	30.3	39.4	38.5	39.2	40.9	38.7	41.2	38.7	40.1	39.2	40.9	40.4	40.6
Primer efficiency values (%)	98	98	100	97	89	99	100	100	93	99	98	97	97

Table S3. Sequence identity among the 13 GRF proteins of tomato.

Protein	A	B	C	D	E	F	G	H	I	J	K	L	M
SlGRF1 (A)	100												
SlGRF2 (B)	73	100											
SlGRF3 (C)	76	76	100										
SlGRF4 (D)	38	39	39	100									
SlGRF5 (E)	31	35	32	35	100								
SlGRF6 (F)	33	33	50	56	41	100							
SlGRF7 (G)	47	47	45	46	48	42	100						
SlGRF8 (H)	53	58	56	47	43	46	50	100					
SlGRF9 (I)	48	50	50	47	40	42	44	86	100				
SlGRF10 (J)	53	52	55	49	43	37	42	48	46	100			
SlGRF11 (K)	52	54	48	58	44	50	44	50	47	53	100		
SlGRF12 (L)	41	45	49	40	42	52	29	43	37	49	57	100	
SlGRF13 (M)	54	44	43	44	38	48	39	41	42	46	49	45	100

Please define lettering in table footer.

Table S4. Putative *cis*-elements of more than 5 bp identified in 13 *GRF* genes from *Solanum lycopersicum* using PlantCARE database (URL).

Commented [I3]: For missing data or NA data, please fill cells with hyphen (i.e., "-")

Formatted Table

Formatted: Highlight

Formatted: MDPI_4.3_table_footer

Formatted: Font: Bold

Formatted: Font: Italic, Not Expanded by / Condensed by

Commented [I4]: Please add URL, for consistency with Figure S3 footer

Formatted: Left: 1.9 cm, Right: 2.5 cm, Top: 2.7 cm, Bottom: 2.7 cm, Width: 29.7 cm, Height: 21 cm

Table S5. Putative functions and cellular localizations of tomato GRF proteins.

Gene name	GO: Molecular function	GO: Biological process	GO: Cellular component
SlGRF1	ATP binding; adenyl ribonucleotide binding, purine ribonucleoside binding, purine ribonucleotide binding, ribonucleotide binding, carbohydrate derivative binding, nucleoside phosphate binding, nucleoside binding, ion and anion binding	Regulation of transcription DNA templated	Nucleus
SlGRF2	ATP binding; adenyl ribonucleotide binding, purine ribonucleoside binding, purine ribonucleotide binding, ribonucleotide binding, carbohydrate derivative binding, nucleoside phosphate binding, nucleoside binding, ion and anion binding	Regulation of transcription	Nucleus
SlGRF3	ATP binding; adenyl ribonucleotide binding, purine ribonucleoside binding, purine ribonucleotide binding, ribonucleotide binding, carbohydrate derivative binding, nucleoside phosphate binding, nucleoside binding, ion and anion binding	Regulation of transcription	Nucleus
SlGRF4	ATP binding; adenyl ribonucleotide binding, purine ribonucleoside binding, purine ribonucleotide binding, ribonucleotide binding, carbohydrate derivative binding, nucleoside phosphate binding, nucleoside binding, ion and anion binding	Regulation of transcription	Nucleus
SlGRF5	ATP binding; adenyl ribonucleotide binding, purine ribonucleoside binding, purine ribonucleotide binding, ribonucleotide binding, carbohydrate derivative binding, nucleoside phosphate binding, nucleoside binding, ion and anion binding	Regulation of transcription	Nucleus
SlGRF6	ATP binding; adenyl ribonucleotide binding, purine ribonucleoside binding, purine ribonucleotide binding, ribonucleotide binding, carbohydrate derivative binding, nucleoside phosphate binding, nucleoside binding, ion and anion binding	Regulation of transcription	Nucleus
SlGRF7	ATP binding; adenyl ribonucleotide binding, purine ribonucleoside binding, purine ribonucleotide binding, ribonucleotide binding, carbohydrate derivative binding, nucleoside phosphate binding, nucleoside binding, ion and anion binding	Regulation of transcription	Nucleus
SlGRF8	ATP binding; adenyl ribonucleotide binding, purine ribonucleoside binding, purine ribonucleotide binding, ribonucleotide binding,	Regulation of transcription	Nucleus

Formatted: Font: 9 pt, Bold

Formatted: MDPI_4.2_table_body

Formatted Table

Formatted: Font: 9 pt

Formatted: MDPI_4.2_table_body

	carbohydrate derivative binding, nucleoside phosphate binding, nucleoside binding, ion and anion binding		
SlGRF9	ATP binding; adenyl ribonucleotide binding, purine ribonucleoside binding, purine ribonucleotide binding, ribonucleotide binding, carbohydrate derivative binding, nucleoside phosphate binding, nucleoside binding, ion and anion binding	Regulation of transcription	Nucleus
SlGRF10	ATP binding; adenyl ribonucleotide binding, purine ribonucleoside binding, purine ribonucleotide binding, ribonucleotide binding, carbohydrate derivative binding, nucleoside phosphate binding, nucleoside binding, ion and anion binding	Regulation of transcription	Nucleus
SlGRF11	ATP binding; adenyl ribonucleotide binding, purine ribonucleoside binding, purine ribonucleotide binding, ribonucleotide binding, carbohydrate derivative binding, nucleoside phosphate binding, nucleoside binding, ion and anion binding	Regulation of transcription	Nucleus
SlGRF12	ATP binding; adenyl ribonucleotide binding, purine ribonucleoside binding, purine ribonucleotide binding, ribonucleotide binding, carbohydrate derivative binding, nucleoside phosphate binding, nucleoside binding, ion and anion binding	Regulation of transcription	Nucleus
SlGRF13	ATP binding; adenyl ribonucleotide binding, purine ribonucleoside binding, purine ribonucleotide binding, ribonucleotide binding, carbohydrate derivative binding, nucleoside phosphate binding, nucleoside binding, ion and anion binding	Regulation of transcription	Nucleus

GO: Define.

Formatted: MDPI_4.3_table_footer, Centered
